# Nasal mucus-derived KLK13 restricts SARS-CoV-2 infection via proteolytic cleavage of spike

**DOI:** 10.1128/mbio.02051-25

**Published:** 2025-10-20

**Authors:** Wenying Cao, Ningze Zheng, Hehe Cao, Ran Chen, Jianheng Chen, Xueyi Deng, Hui Zhang, Shuofeng Yuan, Guigen Zhang

**Affiliations:** 1 Institute of Human Virology, Department of Pathogen Biology and Biosecurity, and Key Laboratory of Tropical Disease Control of Ministry of Education, Zhongshan School of Medicine, Sun Yat-sen University 74644 , Guangzhou, China; 2 State Key Laboratory of Emerging Infectious Diseases, Carol Yu Centre for Infection, Department of Microbiology, School of Clinical Medicine, Li Ka Shing Faculty of Medicine, The University of Hong Kong, Pokfulam, Hong Kong Special Administrative Region, China; Washington University in St. Louis School of Medicine, St. Louis, Missouri, USA

**Keywords:** coronavirus, nasal mucus, restriction factor, KLK13 protease, spike protein

## Abstract

The epithelial cilia are the first line of defense against respiratory pathogens. For the first time, we found that Kallikrein-related peptidase 13 (KLK13), a serine protease expressed in airway ciliated epithelial cells with cell type specificity, was secreted into nasal mucus. KLK13 efficiently cleaved the spike of SARS-CoV-2, resulting in the inhibition of SARS-CoV-2 cell entry and spike protein-mediated cell-cell fusion. Recombinant KLK13 protease efficiently cleaved the spike protein as well as virus particles *in vitro*. Only KLK13, but not other members of the KLK family, specifically cleaved the spike proteins of SARS-CoV-2 as well as other coronaviruses. We also confirmed that endogenous KLK13 stimulated by CRISPR activation (CRISPRa) in A549 cells inhibited SARS-CoV-2 pseudovirus entry. The mRNA level of *KLK13* was stimulated by poly (I:C) in both A549 and HeLa cells, and its expression level was also increased in SARS-CoV-2-infected clinical samples. Recombinant adeno-associated virus packaged KLK13 (AAV-KLK13) reduced SARS-CoV-2 replication in a K18-ACE2 mouse model. Collectively, the nasal mucus-derived KLK13 functions as a scissor of coronaviruses and holds the potential to be further developed as a broad-spectrum antiviral against coronaviruses.

**IMPORTANCE:**

Epithelial cilia directly come into contact with inhaled pathogens. The nasal mucus functions as a formidable barrier against penetration of viral particles. KLK13 is secreted into nasal mucus and efficiently cleaves the spike proteins across different coronavirus species. KLK13-mediated cleavage of spike inhibits SARS-CoV-2 entry and syncytium formation. Intranasally delivered KLK13 also restricts SARS-CoV-2 infection *in vivo*. The finding that KLK13 acts as a scissor of viral spike in nasal mucus paves the way for the development of new antivirals against respiratory viruses.

## INTRODUCTION

Epithelial cilia are the first line of defense against respiratory viruses, such as influenza A viruses, human coronaviruses, and rhinoviruses. Respiratory viruses target the ciliated respiratory epithelial cells at the early stage of infections (1–5). Respiratory epithelial cells are covered by a gel-like layer of mucus, which contains a range of mucin glycoproteins secreted. The heavily glycosylated mucins form a formidable barrier against the penetration of respiratory virus particles. It has been reported that the components of mucus, such as mucins and defensins, restrict respiratory viral infections (6–8).

Coronaviruses (CoVs) are positive-sense single-stranded RNA viruses that are classified into four genera: α-, β-, γ-, and δ-CoVs. In the past 20 years, there have already been three outbreaks caused by β-CoVs, including severe acute respiratory syndrome coronavirus (SARS-CoV) (9–11), Middle East respiratory syndrome coronavirus (MERS-CoV) (12, 13), and severe acute respiratory syndrome coronavirus 2 (SARS-CoV-2) (14, 15). All these viruses placed a substantial burden on global public health, especially SARS-CoV-2, which has caused over seven million deaths. SARS-CoV-2 infection is initiated by binding of the spike protein to its receptor, angiotensin-converting enzyme 2 (ACE2), on the cell surface. After attachment, additional cleavage at the S2′ site by cell surface proteases, such as transmembrane protease serine 2 (TMPRSS2), or by cathepsins in endosomes following endocytosis, releases the fusion peptide and mediates membrane fusion (16, 17). Spike-mediated syncytium formation was observed both *in vitro* and *ex vivo* infected with SARS-CoV, MERS-CoV, or SARS-CoV-2 (18–23). Syncytium formation in the lung tissues of infected patients contributes to SARS-CoV-2 pathogenesis and is a hallmark of severe COVID-19 pathology. Host restriction factors, such as IFN-inducible lymphocyte antigen 6 complex locus E (LY6E), cholesterol 25-hydroxylase (CH25H), inhibited coronavirus infection-associated syncytia (24–26).

Kallikrein-related peptidases (KLKs), a subgroup of serine proteases with diverse physiological functions, have emerged as new regulators of viral infections in recent years. For instance, KLK1, KLK5, and KLK12 have been shown to cleave hemagglutinin proteins of influenza viruses and mediate viral activation (27–29). KLK8 proteolytically processes human papillomaviruses to facilitate viral entry into host cells (30). KLK13 was upregulated in varicella zoster virus-infected keratinocytes and HCoV-HKU1-infected human airway epithelial cells (31, 32). A recent report claimed that KLK13 serves as a priming protease facilitating viral entry during HCoV-HKU1 infection (32). KLK13 is expressed in multiple human tissues and detected in several biological fluids, including cervicovaginal fluid, seminal plasma, and saliva (33, 34). For the first time, we found that KLK13 was secreted into nasal mucus. KLK13 cleaves the SARS-CoV-2 spike protein and antagonizes SARS-CoV-2 infection. The discovery of KLK13 as a scissor of viral spike gave new insights for the development of novel antiviral strategies.

## RESULTS

### KLK13 specifically cleaves the spike of SARS-CoV-2

**Fig 1:**
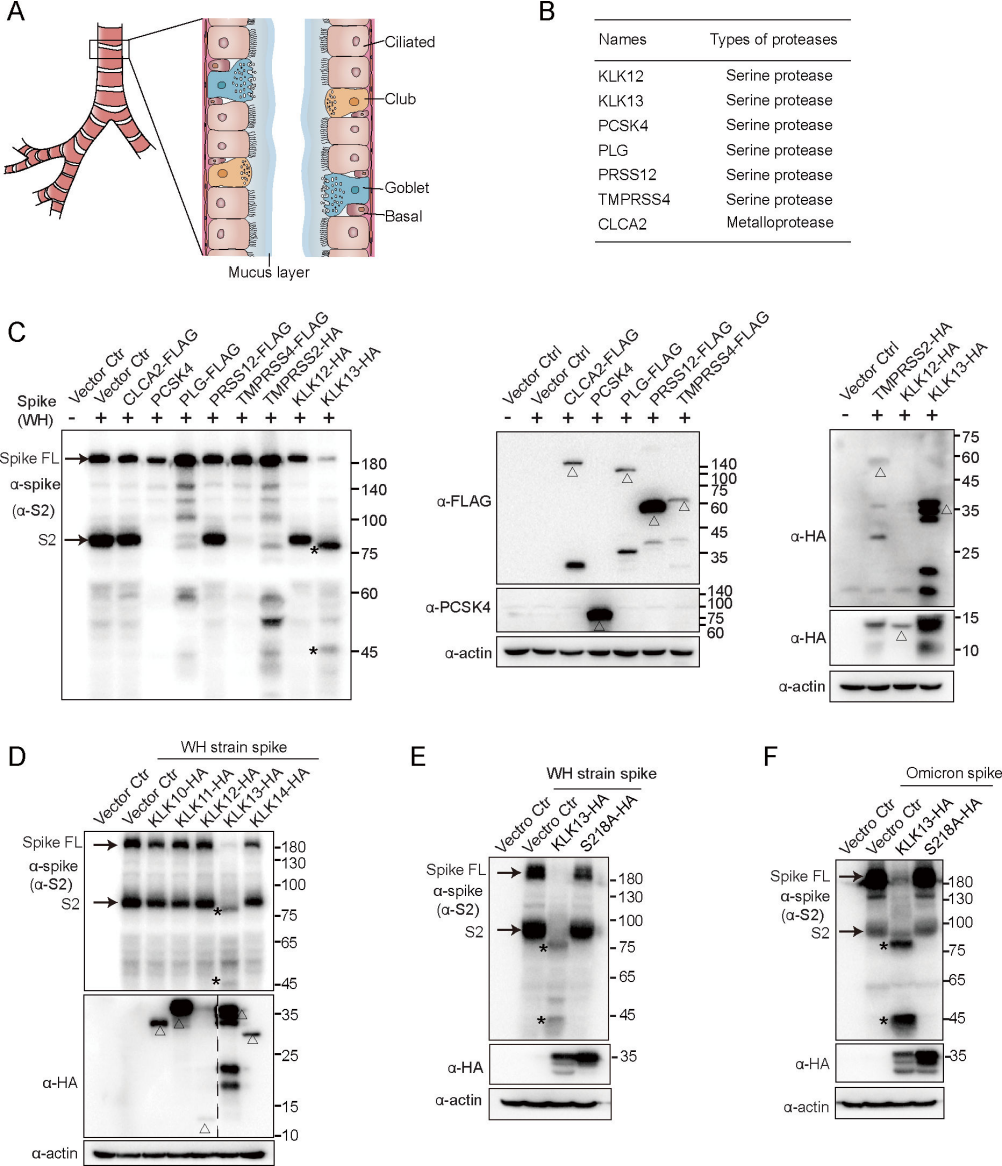
KLK13 specifically cleaved SARS-CoV-2 spike protein. (**A**) Schematic diagram of human trachea. The human upper airway epithelium comprises several types of cells and is covered with a mucus layer. (**B**) The serine proteases and metalloproteases shown in the table were specifically expressed in ciliated respiratory epithelial cells, according to the data sets available from the Human Protein Atlas. (**C**) HEK293T cells were cotransfected with plasmids for SARS-CoV-2 (WH strain) spike together with CLCA2-FLAG, PCSK4, PLG-FLAG, PRSS12-FLAG, TMPRSS4-FLAG, TMPRSS2-HA, KLK12-HA, KLK13-HA, or empty vector. Forty-eight hours later, the cells were lysed and subjected to immunoblotting with specific antibodies. The spike antibody specifically recognizes the S2 region. The arrowheads indicate the spike full-length (FL) and S2 subunit. The asterisks indicate the cleaved fragments of spike protein. The triangles indicate the proteases overexpressed in the cells. Among them, KLK12 was derived from mRNA transcript variant 5 (NM_001370126.1), which encoded a protein with 144 amino acids in length, a short form of KLK12. This experiment was performed in three biological replicates. (**D**) HEK293T cells were cotransfected with plasmids expressing SARS-CoV-2 (WH strain) spike together with HA-tagged KLK10, KLK11, KLK12, KLK13, KLK14, or empty vector. The cells were lysed, followed by immunoblotting analysis. The triangles indicate different members of the KLK family. HEK293T cells were cotransfected with SARS-CoV-2 WH strain (**E**) or Omicron (BA.1) (**F**) spike plasmid together with HA-tagged wild-type KLK13 or KLK13^S218A^ mutant. Forty-eight hours later, the cells were lysed and subjected to immunoblotting analysis. The asterisks indicate the cleaved fragments of spike protein. The arrowheads indicate the spike full-length (FL) and S2 subunit. These experiments were performed in two biological replicates.

By analyzing the public data available from the Human Protein Atlas (https://www.proteinatlas.org/humanproteome/tissue+cell+type/lung), we found that several membrane proteases were expressed in ciliated epithelial cells with high cell type specificity, including the serine proteases TMPRSS4, PRSS12, PCSK4, PLG, KLK12, and KLK13 as well as metalloprotease CLCA2 (Fig. 1A and B). The expression levels of these genes in ciliated respiratory cells were further evaluated using single-cell sequencing data (Fig. S1A). To investigate whether these proteases are involved in SARS-CoV-2 spike cleavage, each of them was cotransfected with SARS-CoV-2 spike expression vector (Wuhan-Hu-1 strain) in HEK293T cells. Among them, PCSK4 and TMPRSS4 dramatically reduced the production of the S2 subunit. In the presence of PLG or TMPRSS2, the spike protein was cleaved, and additional bands were observed, while CLCA2, PRSS12, and KLK12 (a short form) had no such effect. Besides TMPRSS2, TMPRSS4, and plasminogen (PLG) were reported to cleave the SARS-CoV-2 spike (35, 36), which is consistent with our findings. The role of PCSK4 in SARS-CoV-2 spike protein processing and maturation is under investigation. Immunoblotting analysis showed that, in the presence of KLK13, two faster-migrating fragments of the spike protein were detected (Fig. 1C). One fragment was close to, but smaller than, S2, and the other one was slightly larger than 45 kDa. These results indicated that KLK13 cleaves the spike of SARS-CoV-2. Similarly, KLK13 also cleaved the spike of SARS-CoV-2 Omicron BA.1 strain (Fig. S1B). We further investigated whether the other members of the KLK family are able to cleave the spike protein. Strikingly, only KLK13, but not KLK10, KLK11, KLK12, or KLK14, can cleave the spike of SARS-CoV-2 (Fig. 1D; Fig. S1C). To evaluate whether the serine protease activity of KLK13 is necessary for the cleavage of spike, a catalytically inactive mutant KLK13^S218A^ was constructed (37), which was not able to cleave the SARS-CoV-2 spike protein (Fig. 1E through F). Additionally, to analyze whether the cleavage of spike protein by KLK13 is dose-dependent, we co-transfected the spike expression vector with different amounts of KLK13 expression vector in HEK293T cells. As shown in Fig. S2A, the cleaved fragments of spike (above 75 kDa) are increased, along with the increase of KLK13, which confirms that KLK13 cleaves the spike protein in a dose-dependent manner. These results demonstrated that KLK13 specifically cleaves the spike of SARS-CoV-2.

### KLK13 is interferon-stimulated and secreted into nasal mucus

**Fig 2:**
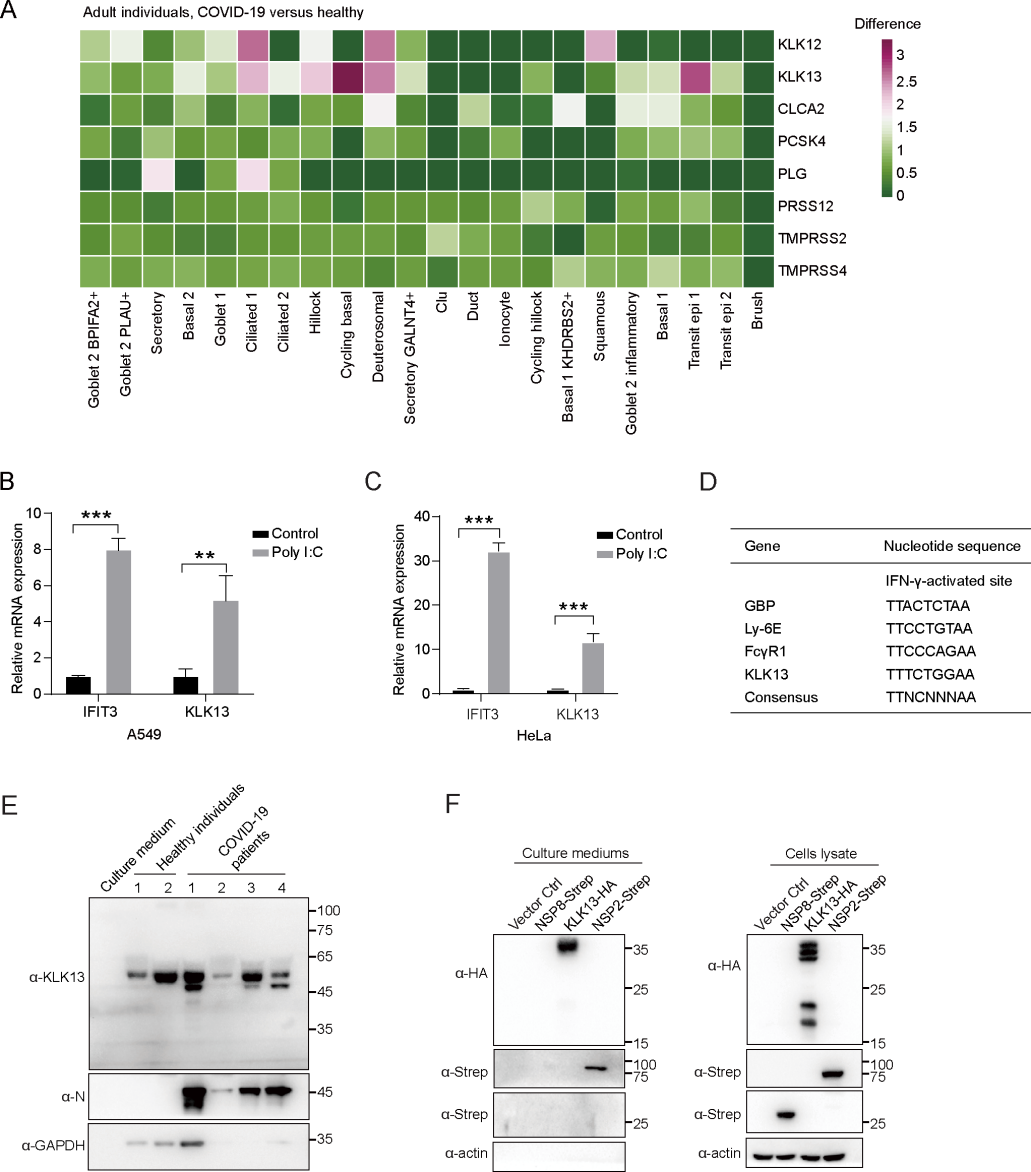
KLK13 is interferon-stimulated and secreted into nasal mucus. (**A**) *KLK13* expression was upregulated in SARS-CoV-2-infected human airway epithelium. Heatmap comparing the expression levels of identified serine proteases and metalloproteases in airway epithelial cells in COVID-19 patients versus healthy individuals. A549 (**C**) or HeLa (**D**) cells were transfected with poly(I:C) (5  µg/mL) or ddH_2_O as a control. Eighteen hours post-transfection, the cells were lysed, followed by qPCR analysis. The *KLK13* mRNA expression level was quantified, while IFIT3 served as a positive control. Data represent three technical replicates, and statistical analysis was conducted with paired Student’s *t*-test, **P < 0.01, ***P < 0.001 (*n* = 3). This experiment was performed in two biological replicates. (**D**) The IFN-γ activation site in the promoter region of *KLK13*. The putative binding sites for STAT1 in the *KLK13* promoter (2000 nt upstream) were predicted by the JASPAR database; a threshold of 0.9 matrix similarity score was applied. (**E**) The nasal mucus collected from two healthy adult individuals and four COVID-19 patients was individually mixed with 2× SDS lysis buffer. The lysates were analyzed by immunoblotting with KLK13-, nucleocapsid-, and GAPDH-specific antibodies, while cell culture medium was used as a control. (**F**) HEK293T cells were transfected with KLK13-HA, SARS-CoV-2 NSP8-Strep, NSP2-Strep, or empty vector, respectively. The culture supernatants and cell lysates were harvested and prepared for immunoblotting analysis.

We analyzed the single-cell sequencing data from both COVID-19 patients and healthy individuals. The expression of *KLK13* in SARS-CoV-2-infected individuals was upregulated compared with that in non-infected individuals (Fig. 2A). To understand whether *KLK13* is an interferon-stimulated gene (ISG), we stimulated both A549 and HeLa cells with poly(I:C), an analog of dsRNA that mimics the molecular pattern associated with viral infection. As shown in Fig. 2B and C, similar to that of *IFIT3*, the mRNA level of *KLK13* was significantly increased in both A549 and HeLa cells upon poly(I:C) stimulation. In addition, the promoter region of *KLK13* was predicted to harbor a putative IFN-γ activation site (Fig. 2D). The IFN-γ activation sites in the promoter regions of *GBP*, *Ly-6E*, and *FcγR1* were shown in parallel (38). These results indicated that *KLK13* is an ISG and upregulated upon SARS-CoV-2 infection. Moreover, since KLK13 was reported to be secreted into several body fluids (33, 34), we therefore tested whether it exists in the nasal mucus. As shown in Fig. 2E, KLK13 was detected in the nasal mucus of both healthy and COVID-19 volunteers, in line with the previous nasal mucus proteomics studies (39–41). We further confirmed that KLK13 was detected in the supernatants of HEK293T cells transfected with *KLK13* cDNA (Fig. 2F). SARS-CoV-2 NSP2 was previously reported to be secreted (42) and was thereby used here as a positive control. The results confirmed that KLK13 is interferon stimulated and can be secreted into nasal mucus.

### Recombinant KLK13 cleaves the spike protein both *in vitro* and *in vivo*

**Fig 3:**
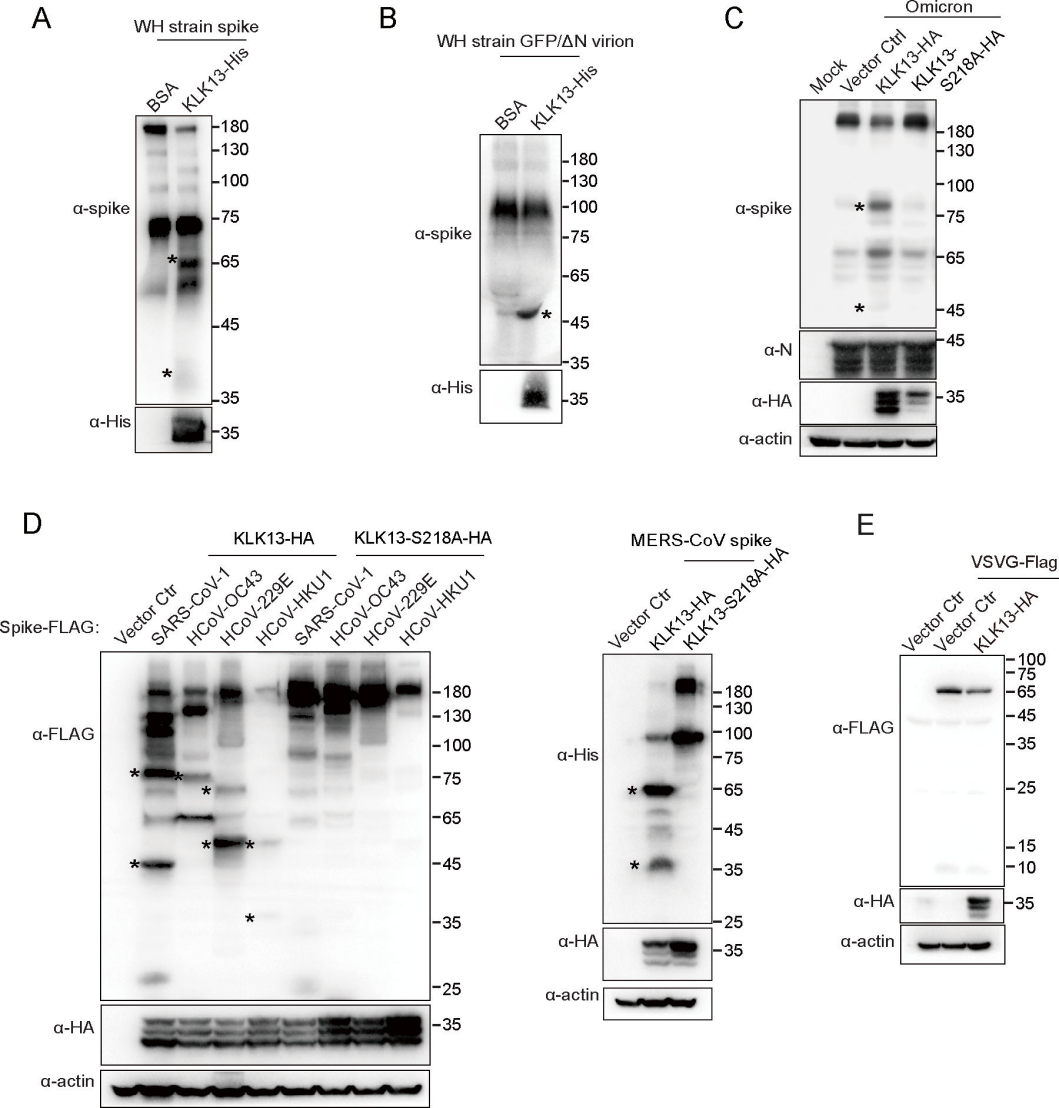
Recombinant KLK13 cleaved both the purified spike protein and the spikes on the virions. The purified SARS-CoV-2 (WH strain) spike (S1 + S2 ECD, 1,193 amino acids) (**A**) or concentrated supernatant containing SARS-CoV-2 (WH strain) GFP/∆N virions (**B**) was incubated with activated KLK13 protein or BSA at 37℃, respectively. Western blotting analysis was performed 3 h post-incubation with spike- and His tag-specific antibodies. This experiment was performed in three biological replicates. (**C**) HEK293T-ACE2 cells were transfected with HA-tagged KLK13, KLK13^S218A^ mutant plasmids, or empty vector for 17 h and infected with SARS-CoV-2 (Omicron strain) for 72 h. The cells were then lysed for immunoblotting analysis with specific antibodies. (**D**) HEK293T cells were cotransfected with plasmids expressing HA-tagged KLK13 (WT) or KLK13^S218A^ together with plasmids expressing FLAG-tagged spikes of SARS-CoV-1, HCoV-OC43, HCoV-229E, or HCoV-HKU1, or His-tagged MERS-CoV spike. After 48 h, the cells were lysed for immunoblotting analysis. The asterisks indicate the cleaved fragments of different spike proteins. (**E**) Plasmid of FLAG-tagged VSV-G was transfected together with KLK13-HA overexpressing vector or empty vector into HEK293T cells, respectively. The cells were lysed for immunoblotting analysis. This experiment was performed in three biological replicates.

To investigate whether KLK13 directly cleaves spike protein *in vitro*, we incubated the purified SARS-CoV-2 (WH strain) spike protein (S1+S2 ECD) with recombinant KLK13 *in vitro*. As shown in Fig. 3A, recombinant KLK13 protease was able to cleave the recombinant spike protein. Since the recombinant spike protein (S1+S2 ECD) consists of 1,193 amino acids, without the transmembrane domain (TMD) and cytoplasmic tail, the cleaved fragments are slightly smaller than those in the full-length spike. We further analyzed whether the recombinant KLK13 protease is capable of cleaving the spikes on the virus particles. The replication-competent SARS-CoV-2 virus-like particles (SARS-CoV-2 GFP/ΔN trVLP) were produced and propagated in the Caco-2-N packaging cells (43). The SARS-CoV-2 virus-like particles were incubated with recombinant KLK13 protease *in vitro*. Indeed, KLK13 protease also cleaved the spikes on the virions (Fig. 3B).

Moreover, we checked the cleavage activity of KLK13 in the context of virus infection. HEK293T-ACE2 cells overexpressing KLK13 were infected with the authentic SARS-CoV-2 virus (Omicron BA.5 strain). Immunoblotting analysis showed that the spike protein was cleaved in SARS-CoV-2-infected cells, as evidenced by the two characteristic bands (Fig. 3C). These results confirmed that KLK13 cleaves both the recombinant spike protein and the spikes on the virions in an enzymatic activity-dependent manner.

### KLK13 cleaves the spikes of coronaviruses across different species

KLK13 hydrolyzed the substrates at basic residues with higher efficiency for Arg (R) and Lys (K) (44). The SARS-CoV-2 spike protein possesses a polybasic cleavage motif, RRAR, and is further processed to the S1 and S2 subunits (22). We generated a spike mutant with the RRAR motif deleted (ΔRRAR) (Fig. S2B). In the presence of KLK13, the upper cleaved fragment close to S2 was not detectable when the RRAR motif was deleted, while the shorter fragment, approximately 45 kDa, was still observed (Fig. S2C). These results indicated that deletion of RRAR affected, but did not completely abolish, KLK13-mediated cleavage of spike.

We further investigated whether KLK13 has a proteolytic effect on the spikes of other coronaviruses. We generated expression vectors for the spikes of different coronaviruses, including SARS-CoV-1, MERS-CoV, HCoV-HKU1, HCoV-229E, and HCoV-OC43. As shown in Fig. 3D, KLK13 cleaved the spike of SARS-CoV-1. The pattern of cleaved fragments is similar to that of SARS-CoV-2 spike. KLK13 efficiently cleaved the spike of MERS-CoV, resulting in two cleaved fragments, which are close to 65 kDa and 35 kDa (Fig. 3D). Strikingly, KLK13 also cleaved the spikes of HCoV-HKU1, HCoV-229E, and HCoV-OC43, although the patterns of cleaved fragments differ from each other. We then investigated whether KLK13 cleaves the glycoproteins of other enveloped viruses. As shown in Fig. 3E, KLK13 was not able to cleave the glycoprotein of vesicular stomatitis virus (VSV). These results indicated that KLK13 is able to cleave the spikes of different coronaviruses, although the cleavage sites are distinctive.

### KLK13 interacts with spike protein and inhibits spike-mediated syncytium formation

**Fig 4:**
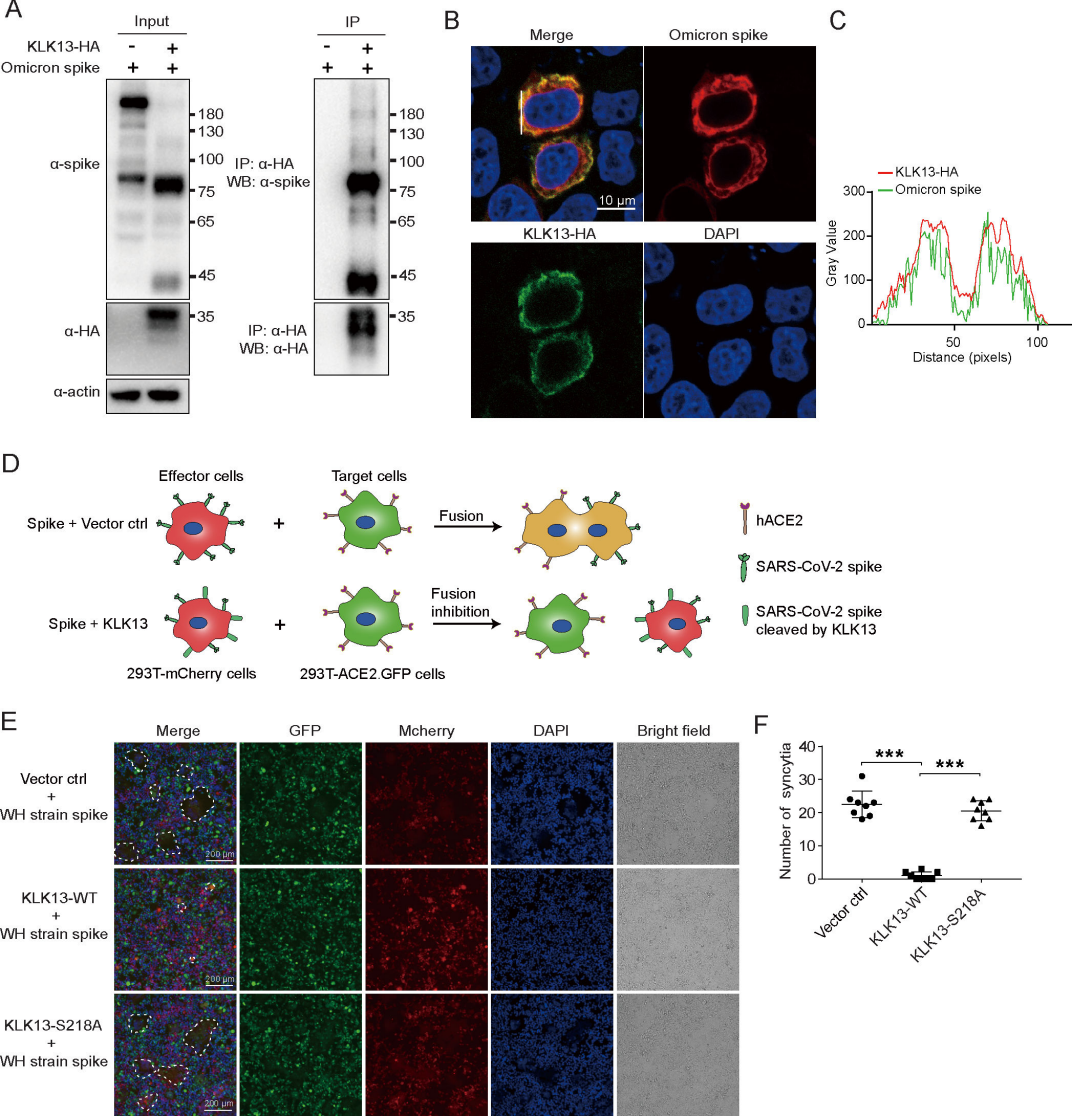
KLK13 interacted with the SARS-CoV-2 spike protein and inhibited spike protein-mediated cell fusion. (**A**) HEK293T cells were cotransfected with an expression plasmid for Omicron (BA.1) spike together with KLK13-HA or empty vector. The cellular lysates were subjected to immunoprecipitation with anti-HA magnetic beads. The precipitates and lysates were blotted with specific antibodies. (**B**) Representative fluorescence images displaying the intracellular location of Omicron (BA.1) spikes and KLK13-HA. HeLa cells were cotransfected with an expression plasmid for Omicron (BA.1) spike together with KLK13-HA or empty vector. Forty-eight hours later, the cells were fixed and subjected to immunofluorescence staining with antibodies against the spike (red) or HA tag (green). DAPI indicated nuclei. (**C**) Colocalization between Omicron (BA.1) spike and exogenous KLK13 along the white line was analyzed using ImageJ software and shown in the histogram. (**D**) Schematic diagram depicting the cell-cell fusion induced by the SARS-CoV-2 spike protein. (**E**) A plasmid expressing SARS-CoV-2 (WH strain) spike was cotransfected, together with KLK13-HA, KLK13^S218A^ mutant, or empty vector into HEK293T-mCherry cells for 5 h. The cells were digested and mixed with HEK293T cells expressing ACE2 and GFP at a ratio of 1:1. After 36 h of coculture, the cells were fixed and stained with DAPI. Immunofluorescence microscopy was used to capture fluorescence and bright field images. The syncytium was depicted by merged red-green color and circled by dashed lines. (**F**) Quantification of the syncytia is shown in (**E**). Syncytium number was pooled from eight microscope fields for each experiment (*n* = 8). Student’s *t*-test was used to perform statistical analysis. ***P < 0.001. These experiments were performed in two biological replicates.

We further performed the co-immunoprecipitation assay to evaluate the protein-protein interaction between KLK13 and SARS-CoV-2 spike. As shown in Fig. 4A, the spike protein was coprecipitated with KLK13. We also performed a complementary co-immunoprecipitation experiment by using IgG as control (Fig. S3A). Consistently, the spike protein was specifically coprecipitated with KLK13. These were also supported by an immunofluorescence assay that KLK13 was colocalized with the spike protein (Fig. 4B and C).

The interaction between the SARS-CoV-2 spike and its receptor ACE2 induces syncytium formation (45–48). We therefore performed a syncytium formation assay to investigate whether KLK13 inhibits SARS-CoV-2-mediated cell-cell fusion (49). We transfected spike and KLK13 vectors into HEK293T-mCherry cells (effector cells). The effector cells were then mixed and co-cultured with HEK293T-ACE2/GFP cells (target cells) (Fig. 4D). As shown in Fig. 4E and F, the SARS-CoV-2 spike protein (WH strain) efficiently induced syncytium formation (top panel), which is consistent with the previous study (45). In the presence of KLK13, the spike-mediated syncytium formation was significantly inhibited (middle panel), while KLK13^S218A^ had no such inhibitory effect (bottom panel). Similarly, KLK13 also blocked SARS-CoV-2 Omicron BA.1 spike-mediated syncytium formation (Fig. S2D and E). Furthermore, we performed a second type of syncytium formation assay by transfecting SARS-CoV-2 spike protein (Wuhan strain or Omicron BA.1 strain), together with KLK13-WT or KLK13^S218A^ expression vectors in HEK293T-ACE2-GFP cells. Consistent with the findings shown in Fig. 4E and F and Fig. S2D and E, KLK13 WT, but not KLK13^S218A^, extensively inhibited SARS-CoV-2 spike-induced syncytium formation (Fig. S3B-C). These results confirmed that KLK13 significantly inhibited spike-mediated syncytium formation.

### Endogenous and secreted KLK13 inhibit SARS-CoV-2 entry

**Fig 5:**
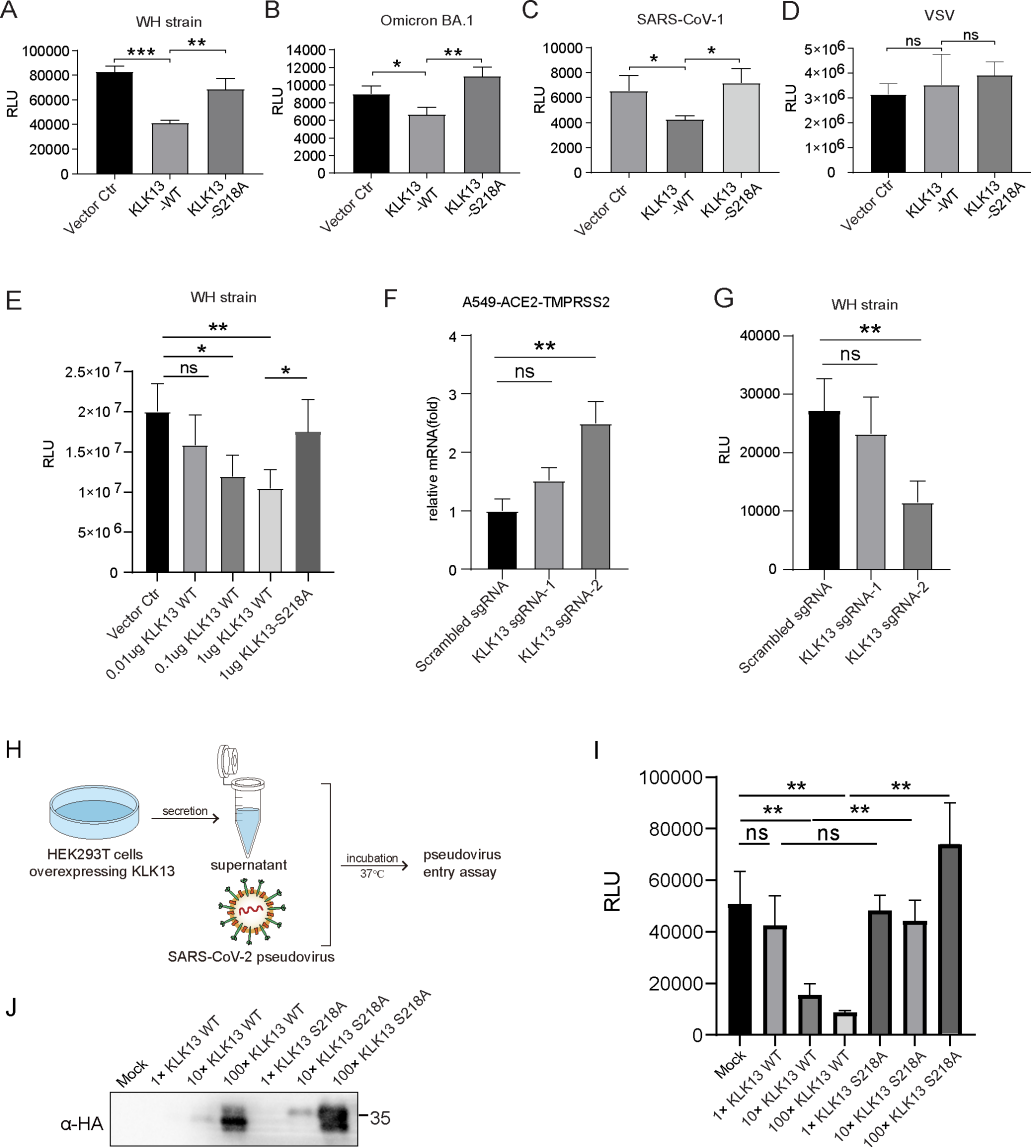
Endogenous and secreted KLK13 inhibit SARS-CoV-2 entry. HEK293T-ACE2 cells were transfected with an expression vector of KLK13-WT, KLK13^S218A^, or empty vector for 24 h, followed by transduction with pseudoviruses bearing SARS-CoV-2 WH strain spike (**A**), Omicron BA.1 spike (**B**), SARS-CoV-1 spike (**C**), or VSV glycoprotein (**D**). Forty-eight hours later, the cells were lysed and subjected to firefly luciferase assays (relative light units, RLU) (*n* = 3). These experiments were performed in three biological replicates. (**E**) HEK293T-ACE2 cells were transfected with different amounts of expression vectors of KLK13-WT, KLK13^S218A^, or empty vector for 24 h, followed by transduction with pseudoviruses. The cells were lysed and subjected to firefly luciferase assays (*n* = 4). (**F**) A549-hACE2-TMPRSS2 cells were transduced with lentiviral vectors expressing CRISPR-dCas9-VPR and KLK13 sgRNAs or scrambled sgRNA. The mRNA expression level of *KLK13* was measured using quantitative PCR (qPCR), which was normalized to the control group. Data are presented as the mean of three technical replicates, and statistical analysis was conducted using a paired Student’s *t*-test, *P < 0.05, **P < 0.01, ***P < 0.001. (*n* = 3). (**G**) The cells described in (**F**) were then transduced with pseudotyped lentiviral particles. The entry of pseudovirus was quantified through measuring the luciferase activity (*n* = 4). This experiment was performed in three biological replicates. (**H**) Schematic representation of pseudovirus entry assay with KLK13-containing supernatants. (**I**) HEK293T cells were transfected with KLK13-WT or KLK13^S218A^ expression vector. The supernatants were collected and concentrated using ultrafiltration tubes. Pseudotyped lentiviral particles were pre-incubated with KLK13 WT- or KLK13^S218A^-containing supernatants at different concentrations at 37℃ overnight. The KLK13-treated pseudoviruses were used to perform the pseudovirus-based entry assays. The luciferase activity was measured (*n* = 3). (**J**) The concentrated supernatants used in (**I**) were blotted with specific antibodies. This experiment was performed in three biological replicates.

We analyzed whether the cleavage of spike protein by KLK13 inhibits SARS-CoV-2 entry. HEK293T-ACE2 cells expressing KLK13 or vector control were transduced with pseudotyped lentiviral particles (WH strain or Omicron BA.1). As presented by the pseudovirus-based entry assays, KLK13-expressing cells showed a lower level of infection than control cells (Fig. 5A and B). Consistently, KLK13 also inhibited SARS-CoV-1 pseudovirus entry (Fig. 5C), but had no effect on the entry of VSV pseudovirus (Fig. 5D). In addition, we investigated whether the inhibition of KLK13 on SARS-CoV-2 pseudovirus entry is dose dependent. Different amounts of KLK13 expression vector were transfected into HEK293T-ACE2 cells. The entry of SARS-CoV-2 pseudovirus decreased, along with the increase of KLK13 (Fig. 5E).

We further analyzed whether endogenous KLK13 plays an inhibitory role in SARS-CoV-2 pseudovirus entry. Since endogenous KLK13 was not detected in different cells, including BEAS-2B, HEK293T, A549, Caco-2, and Calu-3 cells (Fig. S4A and B), we exploited the CRISPR/Cas9-mediated gene activation (referred to as CRISPR activation, CRISPRa) technique to activate the expression of endogenous KLK13 in A549-hACE2-TMPRSS2 cells (50). In this experiment, a CRISPR-dCas9-VPR activation system was used, in which two specific sgRNAs were designed to target the KLK13 gene promoter region, while a scrambled sgRNA was used as a negative control. As shown in Fig. 5F, compared to scrambled sgRNA, sgRNA-2 stimulated KLK13 mRNA expression, whereas sgRNA-1 showed no such effect, which is also considered a control. We then performed the pseudovirus-based entry assay to assess the effect of endogenous KLK13 on viral entry. Consistently, endogenous KLK13 stimulated by CRISPRa inhibited SARS-CoV-2 pseudovirus entry (Fig. 5G).

To study whether secreted KLK13 inhibits SARS-CoV-2 pseudovirus entry, recombinant KLK13 or KLK13^S218A^ protein was concentrated from cell culture supernatants and incubated with pseudotyped lentiviral particles (Fig. 5H). Recombinant KLK13 protein from culture supernatants dose dependently inhibited SARS-CoV-2 pseudovirus entry, while KLK13^S218A^ had no such inhibitory effect (Fig. 5I and J). To further verify if it is the secreted KLK13 that inhibits pseudovirus entry, we designed and synthesized a specific KLK13 peptide inhibitor (Biotin-VRFR-CMK) based on a previous study (51). KLK13-containing supernatants were incubated with KLK13 inhibitor and SARS-CoV-2 pseudoviruses (Fig. S4C). As shown in Fig. S4D, KLK13 inhibitor dose dependently reversed the suppression of KLK13 on SARS-CoV-2 pseudovirus entry. These findings confirm that secreted KLK13 specifically suppresses SARS-CoV-2 entry.

### KLK13 restricts SARS-CoV-2 infection *in vivo*

**Fig 6:**
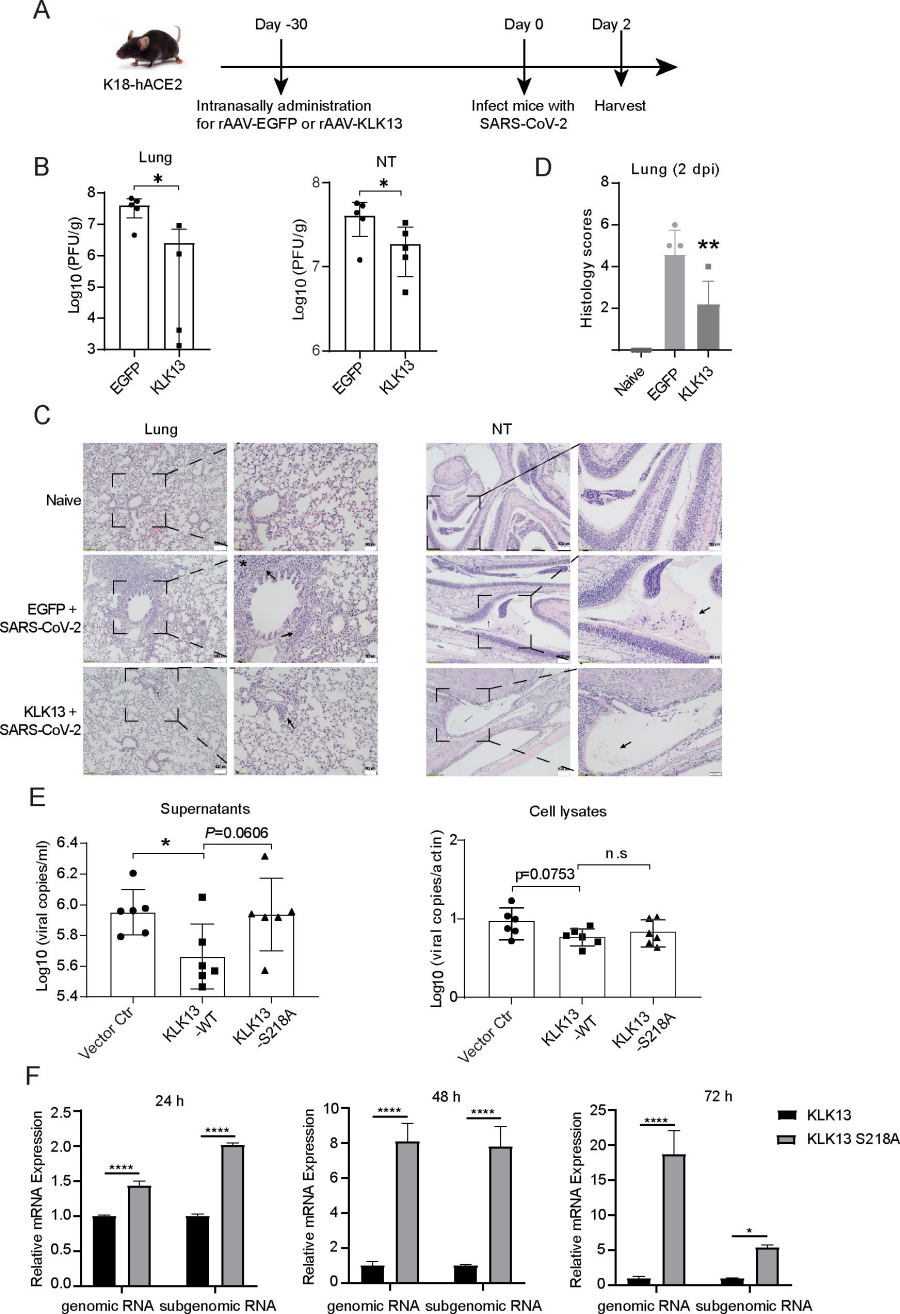
KLK13 restricted the infection of SARS-CoV-2 in a K18-hACE2 mouse model. (**A**) Animal experiment procedures. Male K18-hACE2 transgenic mice were divided into two groups randomly, each group contained five animals. Thirty days before SARS-CoV-2 infection, the mice received rAAV-KLK13 or rAAV-EGFP intranasally. On day 0, all the mice were intranasally challenged with 2 × 10^3^ plaque-forming units SARS-CoV-2 (HKU-001a) after anesthesia. The animals were sacrificed for virological analyses on 2 days post-infection. (**B**) The viral burden in lung and nasal turbinate (NT) was analyzed by plaque assay. Viral titers were quantified as plaque-forming units per gram of tissue (PFU/g) for both lung and nasal turbinate (NT) samples. Student’s *t*-test, *P < 0.05. (*n* = 5). (**C**) The pathological changes of lung and NT were presented. In this experiment, the naïve K18-hACE2 mice were used as controls. Consolidation and immune cell infiltration in the lung were marked by asterisk or arrow, respectively. Exudation and inflammatory cell infiltration in NT were denoted by an arrow. Representative images from five mice in each group were shown at 10× magnification (left, scale bar = 100  µm) and 20× magnification (right, scale bar = 50  µm). (**D**) Histology scores of lung tissues in different groups. Data are mean ± SD. **P < 0.01. (**E**) A549-hACE2-TMPRSS2 cells were transfected with KLK13-WT, KLK13^S218A^ overexpression plasmid, or empty vector, respectively. After 24 h, the cells were infected with SARS-CoV-2 (HKU-001a) at an MOI of 0.01. Forty-eight hours post-infection, the supernatants and cell lysates were harvested and subjected to qPCR assays to quantitate the viral copy numbers. Student’s *t*-test, *P < 0.05, n.s., not significant. (**F**) HEK293T-ACE2 cells were cotransfected with the SARS-CoV-2 nucleocapsid plasmid and KLK13-WT or KLK13^S218A^ for 36 h. The cells were split and infected with SARS-CoV-2 GFP/ΔN for 3 h (MOI of 0.05). Total RNA was extracted 24, 48, and 72 h post-infection (hpi) for RT-qPCR to detect relative levels of SARS-CoV-2 genomic RNA and subgenomic RNA. Data are presented as mean ± SD. *P < 0.05, ****P < 0.0001 (*n* = 3).

KLK13 was expressed in ciliated epithelial cells with cell type specificity. To verify whether KLK13 restricts SARS-CoV-2 infection *in vivo*, we intranasally infected K18-hACE2 transgenic mice with rAAV-KLK13 or rAAV-EGFP (Fig. 6A). Thirty days before SARS-CoV-2 infection, the K18-hACE2 mice were intranasally infected with rAAV-KLK13 or rAAV-EGFP. Each was intranasally challenged with SARS-CoV-2 (HKU-001a strain) on day 0. The animals were sacrificed at 2 dpi (Fig. 6A). The viral burden in the nasal turbinate and lung was measured by plaque assays (Fig. 6B). The results showed that the titer in the lungs of KLK13-expressing mice was reduced, as well as in the nasal turbinate. Moreover, the pathology induced by SARS-CoV-2 in the nasal turbinate and lungs was attenuated by KLK13 (Fig. 6C). In the KLK13 group, exudation and inflammatory cell infiltration in the nasal turbinate were decreased, while consolidation and immune cells were rarely seen in the lung. Histology scores of lung tissues in different groups were also shown (Fig. 6D). These results confirmed that KLK13 restricts SARS-CoV-2 infection in mice. To further investigate the role of KLK13 in viral infection *in vitro*, we overexpressed KLK13 in A549-hACE2-TMPRSS2 cells. Overexpression of KLK13 reduced viral RNA copies in the supernatants (Fig. 6E, left panel), although the viral RNA copies in the cell lysates were not significantly changed (Fig. 6E, right panel). We also performed a time-course infection experiment by using a transcription- and replication-competent SARS-CoV-2 virus-like particle system (SARS-CoV-2 GFP/ΔN trVLP), which enables the complete viral life cycle in HEK293T-ACE2 cells expressing the SARS-CoV-2 nucleocapsid protein (43). Consistently, KLK13-WT, but not KLK13^S218A^, robustly inhibited viral RNA replication 24, 48, and 72 hpi, indicated by the levels of both SARS-CoV-2 genomic and subgenomic RNA (Fig. 6F). Here, the antiviral effect of KLK13 in A549 cells is not as strong as that in HEK293T cells, because the transfection efficiency of KLK13 expression vector in A549 cells is much lower compared to that in HEK293T cells. These results demonstrate that KLK13 exerts antiviral activity against SARS-CoV-2 both *in vivo* and *in vitro*.

## DISCUSSION

Ciliated epithelium is present in the nasal and sinus cavities, as well as the proximal and distal conducting airways. Notably, ciliated epithelial cells are the major targets of respiratory viruses at the early stage of infections. For instance, influenza viruses specifically infected ciliated cells in an *in vitro* model of human ciliated airway epithelia (HAE) (2, 3). Human respiratory syncytial virus (RSV), which causes serious pediatric respiratory disease worldwide, preferentially infects the ciliated cells of the airway epithelium via the apical surface (1). SARS-CoV-1 only infects human HAE derived from nasal and tracheobronchial regions via the apical surface, but not undifferentiated primary epithelial cells (2). The respiratory epithelial cells are covered by a gel-like layer of mucus, which protects against inhaled pathogens. We confirmed that KLK13 was secreted in the nasal mucus of both healthy individuals and COVID-19 patients. MUC5AC and MUC5B, two major secreted airway mucins, are the major components that constitute the airway mucus layer. MUC1, MUC5AC, and MUC5B are known to protect against respiratory viral infections at the respiratory surface (7, 52–54). It seems that the secretion of antiviral components into the mucus is a common strategy to protect against respiratory viral infections. In this study, for the first time, we reported that KLK13 is a new antiviral component secreted into nasal mucus. KLK13 acts as a scissor and cleaves the spikes of different coronaviruses and protects against SARS-CoV-2 infection *in vivo*. Previously, it was reported that TMPRSS4 and plasminogen (PLG) can cleave the SARS-CoV-2 spike (35, 36), which is consistent with our findings (Fig. 1C). Besides, we found that PCSK4 dramatically reduced the production of S1/S2 subunits (Fig. 1C), and the underlying mechanism of which is under investigation.

After cleavage by KLK13, two cleaved fragments of SARS-CoV-2 spike were observed. KLK13 also cleaves the spike of SARS-CoV-1. The pattern of cleaved fragments of SARS-CoV-1 spike is similar to that of SARS-CoV-2. There were two possible cleavage sites in the spike of SARS-CoV-1 and SARS-CoV-2. The upper fragment was not detected when the RRAR motif of spike was deleted in the presence of KLK13 (Fig. S2C). Moreover, KLK13 also cleaved the spikes of MERS-CoV, HCoV-HKU1, HCoV-229E, and HCoV-OC43, although the pattern of cleaved fragments differs from each other. The cleavage sites in these spikes need to be further investigated. It has been reported that KLK13 could cleave the spike of HCoV-HKU1 and serve as a priming protease during HCoV-HKU1 infection (32). It seems that after cleavage by KLK13, the spikes of coronaviruses can either be primed or inactivated. A recent study reported that KLK13, together with KLK12, enhanced SARS-CoV-2 replication; however, the individual role of KLK13 was not fully investigated (55). In our study, both types of syncytium formation assays confirmed that KLK13 WT, but not KLK13^S218A^ mutant, efficiently restricted SARS-CoV-2 spike--mediated cell-cell membrane fusion, which indicates that KLK13 could not be an activator of the SARS-CoV-2 spike protein. The roles of KLK13 in other coronaviral infections, such as MERS-CoV, HCoV-229E, and HCoV-OC43, will be further evaluated both *in vitro* and *in vivo*. The expression of *KLK13* mRNA in various cell lines is very low or undetectable based on analyzing the data from the Human Protein Atlas database (Fig. S4A). KLK13 was not detected in different cells by Western blotting, including A549, Caco-2, BEAS-2B, and Calu-3 cells, although different KLK13 antibodies were used (Fig. S4B). We also performed the mRNA-seq experiment in differentiated primary human nasal epithelial cells under ALI conditions. The mRNA level of *KLK13* was also not high in the differentiated epithelial cells (data not shown). Thereby, we could not perform *KLK13* knockdown or knockout experiments in these cells. Instead, we exploited the CRISPR activation (CRISPRa) technique to stimulate endogenous KLK13 expression in A549-hACE2-TMPRSS2 cells. Previously, we have successfully performed the CRISPR inhibition (CRISPRi) experiments (56). Two sgRNAs targeting the KLK13 gene promoter region were used in this experiment. Scrambled sgRNA was used as a negative control. As shown in Fig. 5G, stimulation of endogenous KLK13 by CRISPR-dCas9-VPR/sgRNA2 inhibits SARS-CoV-2 pseudovirus entry.

Human *KLK13* harbors a SNP (rs34089525) at amino acid position 109, resulting in a His (H) to Tyr (Y) substitution. This SNP is present at 3% frequency in the European populations and 2.4% frequency in the Latin American population, but is nearly absent in Asian populations (Fig. S5A). We introduced this H109Y variant into a KLK13 expression construct. As shown in Fig. S5B and C, both KLK13 WT and KLK13 ^H109Y^ are expressed at similar levels, while the cleavage efficiency of KLK13 ^H109Y^ toward spike is slightly reduced compared with that of KLK13 WT, suggesting that the naturally occurring variants in *KLK13* may possibly affect SARS-CoV-2 infectivity. Currently, we have no epidemiological evidence indicating that the KLK13 ^H109Y^ mutation is associated with the disease severity of COVID-19 patients. Whether this missense mutation is associated with the severity of respiratory infectious diseases in these populations needs further investigation. KLK13 is not sufficient to control virus spread and transmission in the human population. However, the presence of KLK13 in the nasal mucus likely contributes to reducing disease severity via restricting virus infection. Nevertheless, we report that KLK13, a novel restriction factor, is secreted into nasal mucus and inhibits SARS-CoV-2 infection via cleavage of the spike protein (Fig. S5D).

## MATERIALS AND METHODS

### Cell culture

HEK293T cells were purchased from American Type Culture Collection (ATCC). HeLa, BEAS-2B, Caco-2, and A549 cells were obtained from the Cell Bank of Shanghai Institute of Biological Sciences, Chinese Academy of Sciences. A549-hACE2-TMPRSS2 cells were purchased from InvivoGen. Calu-3 and HEK293T-ACE2 cells were kindly provided by Prof. Hui Zhang (57). HEK293T, HeLa, BEAS-2B, and HEK293T-ACE2 cells were maintained in high-glucose DMEM with 10% fetal bovine serum (FBS, VivaCell), while Caco-2 cells were cultured in high-glucose DMEM with 20% FBS. A549 cells were grown in RPMI 1640 medium with 10% FBS, while A549-hACE2-TMPRSS2 cells were maintained in high-glucose DMEM containing 0.5 µg/mL puromycin and 300 µg/mL hygromycin. All the culture media were supplemented with 100 U/mL penicillin and 100 µg/mL streptomycin, and all the cells were cultured at 37°C with 5% CO_2_.

### Virus

SARS-CoV-2 (HKU-001a) was isolated from COVID-19 patients in Hong Kong. The virus was propagated and titrated in Vero E6-TMPRSS2 cells. Virus titration was performed by plaque assay. The experiments involving infectious SARS-CoV-2 were carried out in the Biosafety Level 3 facility at the Department of Microbiology, the University of Hong Kong or Sun Yat-sen University. At the same time, all the protocols were in line with the approved standard operating procedures.

SARS-CoV-2 (WH strain) GFP/∆N was kindly provided by Prof. Qiang Ding (School of Medicine, Tsinghua University) (43). SARS-CoV-2 (WH strain) GFP/∆N was a virus-like particle that was generated through replacement of the nucleocapsid (N) gene in SARS-CoV-2 genomic RNA by a GFP gene. SARS-CoV-2 GFP/∆N could complete its life cycle only in cells expressing the SARS-CoV-2 N protein.

### Reagents

Primary antibodies used in the present study include rabbit anti-KLK13 (NB200-139, Novus), rabbit anti-SARS-CoV-2 spike (28867-1-AP, Proteintech), rabbit anti-SARS nucleocapsid (200-401-A50, ROCKLAND), rabbit anti-PCSK4 (15106-1-AP, Proteintech), rabbit anti-HA-Tag (3724S, Cell Signaling Technology), rabbit anti-IgG control (30000-0-AP, Proteintech), mouse anti-HA-Tag (66006-2-Ig, Proteintech), mouse anti-His-Tag (66005-1-Ig, Proteintech), mouse anti-Strep II-Tag (ABclonal, AE066), mouse anti-β-actin (81115-1-RR, Proteintech), mouse anti-FLAG-Tag (M185-3, MBL), and mouse anti-GAPDH (60004-1-Ig, Proteintech). The KLK13 inhibitor peptide (Biotin-VRFR-CMK) was synthesized by Sangon Biotech (Shanghai, China). The SARS-CoV-2 spike (S1+S2 ECD) recombinant protein (40589-V27B-B, SinoBiological) was ordered.

### Plasmids

Plasmids psPAX2, pHIV-Luciferase, pcDNA3.1-SARS-CoV-2 (WH strain) spike, pcDNA3.1-Omicron (BA.1) spike, pcDNA3.1-MERS-CoV spike-His, pcDNA3.1-HCoV-OC43 spike-FLAG, and pcDNA3.1-HCoV-229E spike-FLAG were kind gifts from Prof. Hui Zhang (Sun Yat-sen University). The plasmids pLVX-SARS-CoV-2 nsp2-Strep, pLVX- SARS-CoV-2 nsp8-Strep, and pLVX-SARS-CoV-2 nucleocapsid-Strep were generously provided by Dr. Nevan J. Krogan (University of California, San Francisco). The plasmids pCMV3-KLK13-HA (HG10199-CY), pCMV3-PCSK4 (HG21911-UT), pCMV3-SARS-CoV-1 spike-FLAG (VG40150-CF), and pCMV3-FLAG-HKU1 spike (VG40021-NF) were purchased from SinoBiological (Beijing, China). The pcDNA3.1-PDCoV spike-HA plasmid was constructed by GENEWIZ (Suzhou, China). The DNA fragment of PDCoV spike-HA was synthesized and subsequently subcloned into the pcDNA3.1 vector using the BamHI and NotI restriction sites. CLCA2, PLG, PRSS12, TMPRSS2, TMPRSS4,, and KLK10-14 cDNAs were ordered from the Suzhou Institute of Biochemistry and Cell Biology and further subcloned into the pLenti-CMV-BSD vector with HA or FLAG tags. Plasmids KLK13 ^S218A^ and KLK13 ^H109Y^ were obtained by using the ClonExpress II One Step Cloning Kit (Vazyme, C112) with pCMV3-KLK13-HA as a template. To generate a vector expressing the spike glycoprotein of VSV, the cDNA of VSV was amplified by PCR and then cloned into pLVX-IRES-ZsGreen1 through the EcoRI and BamHI restriction sites. Omicron spike mutant with RRAR deletion (∆RRAR) was generated according to the protocol of the ClonExpress II One Step Cloning Kit, with wild-type Omicron spike as a template. The sequences of all cloning plasmids were confirmed by Sanger sequencing.

The expression vectors rAAV-KLK13-HA-2A-EGFP and rAAV-KLK13-HA-2A-KLK13 were constructed by BrainVTA (BrainVTA Co., Ltd., Wuhan, China). AAV6 particles encoding human KLK13 or EGFP were also packaged by BrainVTA.

HEK293T cells were seeded one day prior to transfection, and the indicated plasmids were transfected using polyethylenimine (PEI, Sigma) or Fugene (E2311, Promega) following the manufacturer’s instructions.

### CRISPR activation

The plasmids Lenti-SAM-v2-puro (Addgene, 92062) and Lenti-MS2-P65-HSF1 Hygro (Addgene, 61426) were obtained from Addgene. The sgRNAs targeting the promoter regions of KLK13 were synthesized and cloned into the BsmbI site of the Lenti-SAM-v2-puro plasmid. Scrambled sgRNA was used as a negative control.

HEK293T cells were cotransfected with plasmids expressing sgRNA or P65, psPAX2, and pMD2.G at a ratio of 4:3:1.2 to generate lentiviruses. The culture medium was replaced with fresh medium 6 h after transfection, and lentivirus supernatants were further collected at 48 h, filtered using a 0.45 µm filter, and stored at −80°C. A549-hACE2-TMPRSS2 cells were then transduced with both lentiviral vectors that express sgRNA and MS2-P65-HSF1 Hygro. The mRNA expression levels of *KLK13* were determined by quantitative real-time PCR to detect the activation efficiency.

The following sgRNA primers were used in this study:

(1) KLK13#1: 5’-GGCCACATGGCTCCGGGATC-3’;

(2) KLK13#2: 5’-GGGTGCAGTGGCGAGGTGGG-3’;

(3) scrambled sgRNA: 5′-AAGATGAAAGGAAAGGCGTT-3′.

### Animal experiments

After obtaining consent from the Committee on the Use of Live Animals in Teaching and Research (CULATR) of the University of Hong Kong, all the animal experiments were carried out in biosafety level 3 animal facilities and performed in accordance with the standard operating procedures as well as the NIH Guide for Care and Use of Laboratory Animals. K18-hACE2 transgenic mice were housed and bred at the Center for Comparative Medicine Research (CCMR), affiliated with the University of Hong Kong. The mice were maintained in BSL-2 housing with food and water freely and were delivered at 6 to 8 weeks of age. The animals were transferred to a BSL-3 animal facility for virus challenge. All the rooms housing animals were held at 25°C and 50% humidity.

Four- to six-week-old male Balb/c mice were randomly divided into two groups (*n* = 5 per group): the rAAV-EGFP group and rAAV-KLK13 group. Thirty days before viral infection, each mouse received either 5 × 10^5^ vg of rAAV-EGFP or rAAV-KLK13 through intranasal administration (20 µL per mouse). Thirty days post-inoculation, all mice were anesthetized with ketamine and xylazine, then intranasally infected with 2 × 10^3^ plaque-forming units (PFU) of SARS-CoV-2 (HKU-001a) per mouse. Two days post-challenge, all mice were euthanized for tissue collection. Half of the nasal turbinate and right lung homogenate were used for viral load determination via plaque assay, while the remaining nasal turbinate and left lung were preserved for histological analysis.

### Virus titration by plaque assays

For viral titer determination, harvested nasal turbinates and lung tissues were homogenized in 1 mL DMEM using a Tissue Lyzer II (Qiagen, Germany). Following centrifugation, supernatants were tenfold serially diluted and inoculated onto Vero E6-TMPRSS2 cell monolayers in 24-well plates. After 2 h, the cell medium was replaced with 1% low-melting agarose in DMEM containing 1% FBS. Forty-eight hours post-inoculation, cells were fixed with 4% paraformaldehyde and stained with 0.5% crystal violet in 25% ethanol/distilled water for plaque visualization. Viral titers were quantified as plaque-forming units per gram of tissue (PFU/g) for both lung and nasal turbinate (NT) samples.

### Histology and scoring

Lung tissues were collected and fixed in 10% neutral-buffered formalin. Nasal turbinates underwent decalcification in 10% formic acid for seven days before processing with a TP1020 Leica semi-enclosed benchtop tissue processor. Tissue sections were stained with Gill’s hematoxylin and eosin-Y for H&E staining. Images were acquired using an Olympus BX53 light microscope.

For semi-quantitative histological scoring, blinded evaluation was performed to assess pathological changes in bronchioles, alveoli, and blood vessels using the following criteria: For bronchioles:

0  =  normal structure;
1  =  mild peribronchiolar infiltration;
2  =  peribronchiolar infiltration plus epithelial cell death;
3  =  score 2 plus intrabronchiolar wall infiltration and epithelium desquamation.

For alveoli:

0  =  normal structure;
1  =  alveolar wall thickening and congestion;
2  =  focal alveolar space infiltration or exudation;
3  =  diffuse alveolar space infiltration or exudation or hemorrhage.

For blood vessels:

0  =  normal structure;
1  =  mild perivascular edema or infiltration;
2  =  vessel wall infiltration;
3  =  severe endothelium infiltration.

Data are presented as mean ± SD (*n* = 5). Statistical significance was determined by one-way ANOVA with Tukey’s multiple comparison test for intergroup comparisons. All images represent data from two independent *in vivo* experiments, with each data point representing one biological replicate. Significance levels are denoted as: *P < 0.05; **P < 0.01; ***P < 0.001; ****P < 0.0001; n.s. = not statistically significant.

### Pseudovirus packaging and pseudovirus entry assay

To obtain pseudoviruses bearing VSV glycoprotein, SARS-CoV-1 spike, SARS-CoV-2 (WH strain) spike, or Omicron (BA.1) spike, HEK293T cells were cotransfected with plasmids encoding VSV glycoprotein, SARS-CoV-1 spike, SARS-CoV-2 spike, or Omicron spike along with psPAX2 and pHIV-Luciferase at a ratio of 15:10:10. The medium was refreshed at 6 h post-transfection. Supernatants containing viral particles were harvested 48 h later and subsequently filtered using a 0.45 µm filter and frozen at −80°C.

To perform pseudovirus entry assays, HEK293T-ACE2 cells were seeded in 48-well plates in triplicate and inoculated with pseudovirus the following day. Six hours after transduction, the medium was replaced, and the cells were cultured for another 48 h. The cells were lysed with lysis buffer and subjected to the detection of firefly luciferase signal through a luminometer.

### Immunofluorescence

HEK293T cells were seeded in 24-well plates with cell-climbing slices at a density of 10 × 10^4^ and cultured overnight. The Omicron spike plasmid was transfected into HEK293T cells along with the KLK13-HA plasmid. At 36 h post-transfection, the cells were fixed in 4% paraformaldehyde for 10 min and then permeabilized in 0.2% Triton X-100 in PBS for 10 min at room temperature. Following blocking in 1% BSA diluted in PBS at 37°C for 1 h, the sample was incubated with SARS-CoV-2 spike- or HA tag-specific primary antibody at 37°C for 1 h. After washing three times with PBS to remove unbound primary antibody, goat anti-mouse FITC (015-090-050, Jackson) and goat anti-rabbit Alexa Fluor 568 secondary antibodies (A11036, Invitrogen) in 1% BSA were added and incubated at 37°C for 1 h. The sample was washed three times with PBS and then incubated with DAPI diluted in PBS at room temperature for 7 min. Afterward, the cell-climbing slice was mounted on microscope slides with antifade mounting medium (H-1000-10, Vector). Fluorescence images were captured using a Nikon C2 confocal microscope (Nikon Eclipse Ni-E), and colocalization analysis was performed using ImageJ software (ImageJ_v1.8.0) with the Plot Profile function.

### Cell-cell fusion assay

HEK293T cells stably expressing mCherry were cotransfected with SARS-CoV-2 (WH strain) spike or (BA.1) spike expression plasmids, together with plasmid of KLK13-HA, KLK13^S218A^, or an empty vector, which served as effector cells. At 5 h post-transfection, the cells were detached and mixed at a 1:1 ratio with HEK293T cells stably expressing GFP and ACE2, which were used as target cells. Thirty-six hours after coculture, the mixed cells were fixed with 4% paraformaldehyde and subsequently stained with DAPI. Fluorescent and bright-field images were captured using a fluorescence microscope (Olympus). The number of syncytia, indicated by merged green-red color, was counted and analyzed from eight fields per experiment.

### RNA extraction, cDNA preparation, and quantitative real-time PCR

HeLa cells or A549 cells were seeded into 12-well plates at a density of 3 × 10^5^ and cultured overnight. Both cell lines were transfected with poly(I:C) using FuGENE HD transfection reagents, with ddH_2_O as a negative control. Total RNA in cells was extracted at 18 h post-transfection using MagZol Reagent (Magen) following the manufacturer’s instructions. The concentrations of purified RNA were measured using a NanoDrop spectrophotometer, and 1 µg of total RNA was used for reverse transcription with HiScript II Q RT SuperMix for qPCR (+gDNA wiper) (Vazyme, China). Quantitative real-time PCR was performed by using ChamQ Universal SYBR qPCR Master Mix (Vazyme, China) to analyze the mRNA expression level of KLK13 in each sample, with IFIT3 serving as a positive control. To quantify the relative expression level of target genes, the housekeeping gene GAPDH served as a reference for normalization. Three technical replicates in each group were adopted in this experiment.

The following primers were used in this study for qPCR:

KLK13-Fwd: 5′-CAGCCCCCAGGTGAATTAC-3′;

KLK13-Rwd: 5′-CAGGAGACGATGCCATACAGT-3′;

IFIT3-Fwd: 5′-GAAGAAATGAAAGGGCGAAGG-3′;

IFIT3-Rwd: 5′-AGGACATCTGTTTGGCAAGGAG-3′;

GAPDH-Fwd: 5′-TGCACCACCAACTGCTTAGC-3′;

GAPDH-Rwd: 5′-GGCATGGACTGTGGTCATGAG-3′;.

SARS-CoV-2 genomic RNA-Fwd: 5′-AGAAGATTGGTTAGATGATGATAGT-3′;

SARS-CoV-2 genomic RNA-Rwd: 5′-TTCCATCTCTAATTGAGGTTGAACC-3′;

SARS-CoV-2 subgenomic RNA-Fwd: 5′-CTTCCCTCAGTCAGCACCTC-3′;

SARS-CoV-2 subgenomic RNA-Rwd: 5′-AACCAGTGTGTGCCATTTGA-3′;

### Coimmunoprecipitation

HEK293T cells were seeded in 6-well plates at a density of 3 × 10^5^ and cultured overnight. The SARS-CoV-2 WH strain or Omicron (BA.1) spike plasmid was transfected into HEK293T cells along with KLK13-HA or an empty vector for 48 h. The cells were lysed in 500 µL TBST lysis buffer (20 mM Tris-HCl pH 7.4, 1 mM EDTA, 150 mM NaCl, 1% Triton X-100), containing 1× proteinase inhibitor cocktail, among which 50 µL of lysate was mixed with 4× loading buffer and then denatured at 98°C for 10 min, while 450 µL of lysate was incubated with 20 µL of anti-HA magnetic beads (Pierce, 88836) or 20 µL protein A/G agarose beads conjugated with normal IgG (as a control) or specific IgG against HA on a rolling platform at 4°C overnight. The beads were separated using a magnetic stand or centrifugation and washed six times with TBST lysis buffer. Proteins bound to beads were eluted by mixing with 4× loading buffer and boiling at 98°C for 10 min. Both cell lysate (input) and immunoprecipitated (IP) samples were subsequently used for immunoblotting analysis.

### Nasal mucus collection and analysis

At enrollment, nasal mucus of volunteers was collected in 15 mL centrifuge tubes and placed on ice immediately. The mucus was mixed with 1% SDS lysis buffer (1% SDS, 50 mM Tris-HCl pH 8.1, 10 mM EDTA pH 8, 1 mM Phenylmethylsulfonylfluoride) containing 2× proteinase inhibitor cocktail at a ratio of 1:1 and further sonicated. The cellular debris in the lysate was removed by centrifugation at 12,000 × *g* at 4°C for 10 min. The concentration of total protein in lysate was determined using a BCA Protein Assay Kit (23227, Thermo Scientific). In each sample, 18 µg of protein was mixed with 4× loading buffer and denatured at 98°C for 10 min and subjected to immunoblotting analysis.

### Immunoblotting

Cells were harvested and lysed in 1× SDS lysis buffer (containing 1× proteinase inhibitor cocktail), while culture media were mixed with 4× loading buffer (containing 4× proteinase inhibitor cocktail). All the samples were denatured at 98°C for 10 min. Proteins were separated by SDS-PAGE and then transferred to PVDF membranes (Merck Millipore). After blocking in 5% skim milk for 1 h, the membranes were incubated with specific primary antibodies on a rolling platform at 4°C overnight. Following three washes with PBST, the membranes were incubated with HRP-conjugated secondary antibodies (115-035-003, Jackson) at room temperature for 1 h. After washing three times with PBS, chemiluminescent images were captured using an image developer (ChemiDoc XRS+) with chemiluminescent HRP substrate (WBKLS0500, Merck).

### SARS-CoV-2 spike cleavage experiment *in vitro*

The recombinant human KLK13 protein (10199-H08H, SinoBiological) is in the form of proenzyme and needed to be activated by lysyl endopeptidase (JP05061, Wako). The lyophilized powder of KLK13 protein and lysyl endopeptidase was diluted in activation buffer (0.1 M Tris, pH 8.0) to the final concentration of 100 µg/mL and 0.8 µg/mL, respectively. The activation assay was conducted by adding 1 µL lysyl endopeptidase to 40 µL of KLK13 protein solution, followed by incubation at 37℃ for 30 min. For the *in vitro* SARS-CoV-2 spike cleavage experiment, the SARS-CoV-2 spike protein (40589-V27B-B, SinoBiological) was firstly diluted in reaction buffer (50 mM tris, pH 7.5) to the final concentration of 25 µg/mL. Then, 10 µL of activated KLK13 protein or BSA (100 µg/mL) solution was fully mixed with 40 µL of SARS-CoV-2 spike protein, respectively, and incubated at 37℃ for 3 h. The samples were mixed with 4× loading buffer and then denatured at 98℃ for 10 min and subjected to immunoblotting analysis.

### scRNA-seq data analysis

All data sets used for expression analysis originated from public data sets. To determine the expression levels of identified serine proteases and metalloproteases across various cell types of the human airway, the human airway single-cell RNA-seq data were downloaded from Synapse (https://accounts.synapse.org/) (accession code: EGAS00001004344). The single-cell RNA-seq data from healthy individuals and SARS-CoV-2-infected patients were available at a web portal (https://covid19cellatlas.org) and used for differential expression analysis of identified genes in specific human airway cell types. Data processing and plotting were conducted in R software.

### Statistical analysis

All data are presented as the mean ± SD. Comparisons were performed by using two-tailed Student’s unpaired *t* tests. Differences between two groups were considered statistically significant when *P* < 0.05.

## SUPPLEMENTAL MATERIAL

Fig. S1 mbio.02051-25-s0001.tif KLK13 cleaves the spike of SARS-CoV-2 (BA.1 strain).

Fig. S2 mbio.02051-25-s0002.tif Deletion of RRAR affected KLK13-mediated cleavage of SARS-CoV-2 spike.

Fig. S3 mbio.02051-25-s0003.tif KLK13 interacted with the SARS-CoV-2 spike protein and inhibited spike protein-mediated cell-cell fusion.

Fig. S4 mbio.02051-25-s0004.tif Low expression of KLK13 in different cell lines.

Fig. S5 mbio.02051-25-s0005.tif KLK13 polymorphism H109Y slightly reduced the cleavage efficiency on SARS-CoV-2 spike.

Legends mbio.02051-25-s0006.docx Supplemental figure legends.

ASM does not own the copyrights to Supplemental Material that may be linked to, or accessed through, an article. The authors have granted ASM a non-exclusive, world-wide license to publish the Supplemental Material files. Please contact the corresponding author directly for reuse.

## References

[1] Zhang L, Peeples ME, Boucher RC, Collins PL, Pickles RJ. 2002. Respiratory syncytial virus infection of human airway epithelial cells is polarized, specific to ciliated cells, and without obvious cytopathology. J Virol 76:5654–5666. doi:10.1128/jvi.76.11.5654-5666.2002 11991994 PMC137037

[2] Sims AC, Baric RS, Yount B, Burkett SE, Collins PL, Pickles RJ. 2005. Severe acute respiratory syndrome coronavirus infection of human ciliated airway epithelia: role of ciliated cells in viral spread in the conducting airways of the lungs. J Virol 79:15511–15524. doi:10.1128/JVI.79.24.15511-15524.2005 16306622 PMC1316022

[3] Matrosovich MN, Matrosovich TY, Gray T, Roberts NA, Klenk HD. 2004. Human and avian influenza viruses target different cell types in cultures of human airway epithelium. Proc Natl Acad Sci U S A 101:4620–4624. doi:10.1073/pnas.0308001101 15070767 PMC384796

[4] Ahn JH, Kim J, Hong SP, Choi SY, Yang MJ, Ju YS, Kim YT, Kim HM, Rahman MDT, Chung MK, Hong SD, Bae H, Lee CS, Koh GY. 2021. Nasal ciliated cells are primary targets for SARS-CoV-2 replication in the early stage of COVID-19. J Clin Invest 131:e148517. doi:10.1172/JCI148517 34003804 PMC8245175

[5] Wu CT, Lidsky PV, Xiao Y, Cheng R, Lee IT, Nakayama T, Jiang S, He W, Demeter J, Knight MG, Turn RE, Rojas-Hernandez LS, Ye C, Chiem K, Shon J, Martinez-Sobrido L, Bertozzi CR, Nolan GP, Nayak JV, Milla C, Andino R, Jackson PK. 2023. SARS-CoV-2 replication in airway epithelia requires motile cilia and microvillar reprogramming. Cell 186:112–130. doi:10.1016/j.cell.2022.11.030 36580912 PMC9715480

[6] Holly MK, Diaz K, Smith JG. 2017. Defensins in viral infection and pathogenesis. Annu Rev Virol 4:369–391. doi:10.1146/annurev-virology-101416-041734 28715972

[7] Chatterjee M, Huang LZX, Mykytyn AZ, Wang C, Lamers MM, Westendorp B, Wubbolts RW, van Putten JPM, Bosch B-J, Haagmans BL, Strijbis K. 2023. Glycosylated extracellular mucin domains protect against SARS-CoV-2 infection at the respiratory surface. PLoS Pathog 19:e1011571. doi:10.1371/journal.ppat.1011571 37561789 PMC10464970

[8] Chatterjee M, van Putten JPM, Strijbis K. 2020. Defensive properties of mucin glycoproteins during respiratory infections—relevance for SARS-CoV-2. mBio 11:e02374-20. doi:10.1128/mBio.02374-20 33184103 PMC7663010

[9] Drosten C, Günther S, Preiser W, van der Werf S, Brodt H-R, Becker S, Rabenau H, Panning M, Kolesnikova L, Fouchier RAM, et al.. 2003. Identification of a novel coronavirus in patients with severe acute respiratory syndrome. N Engl J Med 348:1967–1976. doi:10.1056/NEJMoa030747 12690091

[10] Ksiazek TG, Erdman D, Goldsmith CS, Zaki SR, Peret T, Emery S, Tong S, Urbani C, Comer JA, Lim W, et al.. 2003. A novel coronavirus associated with severe acute respiratory syndrome. N Engl J Med 348:1953–1966. doi:10.1056/NEJMoa030781 12690092

[11] Peiris JSM, Yuen KY, Osterhaus ADME, Stöhr K. 2003. The severe acute respiratory syndrome. N Engl J Med 349:2431–2441. doi:10.1056/NEJMra032498 14681510

[12] Zaki AM, van Boheemen S, Bestebroer TM, Osterhaus ADME, Fouchier RAM. 2012. Isolation of a novel coronavirus from a man with pneumonia in Saudi Arabia. N Engl J Med 367:1814–1820. doi:10.1056/NEJMoa1211721 23075143

[13] Wit E, Doremalen N, Falzarano D, Munster VJ. 2016. SARS and MERS: recent insights into emerging coronaviruses. Nat Rev Microbiol 14:523–534. doi:10.1038/nrmicro.2016.81 27344959 PMC7097822

[14] Zhu N, Zhang D, Wang W, Li X, Yang B, Song J, Zhao X, Huang B, Shi W, Lu R, Niu P, Zhan F, Ma X, Wang D, Xu W, Wu G, Gao GF, Tan W, China Novel Coronavirus I, Research T. 2020. A novel coronavirus from patients with pneumonia in China, 2019. N Engl J Med 382:727–733. doi:10.1056/NEJMoa2001017 31978945 PMC7092803

[15] Zhou P, Yang XL, Wang XG, Hu B, Zhang L, Zhang W, Si HR, Zhu Y, Li B, Huang CL, et al.. 2020. A pneumonia outbreak associated with a new coronavirus of probable bat origin. Nature 579:270–273. doi:10.1038/s41586-020-2012-7 32015507 PMC7095418

[16] Bestle D, Heindl MR, Limburg H, Van Lam van T, Pilgram O, Moulton H, Stein DA, Hardes K, Eickmann M, Dolnik O, Rohde C, Klenk H-D, Garten W, Steinmetzer T, Böttcher-Friebertshäuser E. 2020. TMPRSS2 and furin are both essential for proteolytic activation of SARS-CoV-2 in human airway cells. Life Sci Alliance 3:e202000786. doi:10.26508/lsa.202000786 32703818 PMC7383062

[17] Zhao MM, Yang WL, Yang FY, Zhang L, Huang WJ, Hou W, Fan CF, Jin RH, Feng YM, Wang YC, Yang JK. 2021. Cathepsin L plays a key role in SARS-CoV-2 infection in humans and humanized mice and is a promising target for new drug development. Signal Transduct Target Ther 6:134. doi:10.1038/s41392-021-00558-8 33774649 PMC7997800

[18] Franks TJ, Chong PY, Chui P, Galvin JR, Lourens RM, Reid AH, Selbs E, Mcevoy CPL, Hayden CDL, Fukuoka J, Taubenberger JK, Travis WD. 2003. Lung pathology of severe acute respiratory syndrome (SARS): a study of 8 autopsy cases from Singapore. Hum Pathol (N Y) 34:743–748. doi:10.1016/S0046-8177(03)00367-8 PMC7119137 14506633

[19] Matsuyama S, Nagata N, Shirato K, Kawase M, Takeda M, Taguchi F. 2010. Efficient activation of the severe acute respiratory syndrome coronavirus spike protein by the transmembrane protease TMPRSS2. J Virol 84:12658–12664. doi:10.1128/JVI.01542-10 20926566 PMC3004351

[20] Qian Z, Dominguez SR, Holmes KV. 2013. Role of the spike glycoprotein of human middle east respiratory syndrome coronavirus (MERS-CoV) in virus entry and syncytia formation. PLoS One 8:e76469. doi:10.1371/journal.pone.0076469 24098509 PMC3789674

[21] Chan JF-W, Chan K-H, Choi GK-Y, To KK-W, Tse H, Cai J-P, Yeung ML, Cheng VC-C, Chen H, Che X-Y, Lau SK-P, Woo PC-Y, Yuen K-Y. 2013. Differential cell line susceptibility to the emerging novel human betacoronavirus 2c EMC/2012: implications for disease pathogenesis and clinical manifestation. J Infect Dis 207:1743–1752. doi:10.1093/infdis/jit123 23532101 PMC7107374

[22] Hoffmann M, Kleine-Weber H, Pöhlmann S. 2020. A multibasic cleavage site in the spike protein of SARS-CoV-2 is essential for infection of human lung cells. Mol Cell 78:779–784. doi:10.1016/j.molcel.2020.04.022 32362314 PMC7194065

[23] Xu Z, Shi L, Wang Y, Zhang J, Huang L, Zhang C, Liu S, Zhao P, Liu H, Zhu L, Tai Y, Bai C, Gao T, Song J, Xia P, Dong J, Zhao J, Wang FS. 2020. Pathological findings of COVID-19 associated with acute respiratory distress syndrome. Lancet Respir Med 8:420–422. doi:10.1016/S2213-2600(20)30076-X 32085846 PMC7164771

[24] Pfaender S, Mar KB, Michailidis E, Kratzel A, Boys IN, V’kovski P, Fan W, Kelly JN, Hirt D, Ebert N, et al.. 2020. LY6E impairs coronavirus fusion and confers immune control of viral disease. Nat Microbiol 5:1330–1339. doi:10.1038/s41564-020-0769-y 32704094 PMC7916999

[25] Wang S, Li W, Hui H, Tiwari SK, Zhang Q, Croker BA, Rawlings S, Smith D, Carlin AF, Rana TM. 2020. Cholesterol 25-hydroxylase inhibits SARS-CoV-2 and other coronaviruses by depleting membrane cholesterol. EMBO J 39:e106057. doi:10.15252/embj.2020106057 32944968 PMC7537045

[26] Zang R, Case JB, Yutuc E, Ma X, Shen S, Gomez Castro MF, Liu Z, Zeng Q, Zhao H, Son J, et al.. 2020. Cholesterol 25-hydroxylase suppresses SARS-CoV-2 replication by blocking membrane fusion. Proc Natl Acad Sci U S A 117:32105–32113. doi:10.1073/pnas.2012197117 33239446 PMC7749331

[27] Hamilton BS, Whittaker GR. 2013. Cleavage activation of human-adapted influenza virus subtypes by kallikrein-related peptidases 5 and 12. J Biol Chem 288:17399–17407. doi:10.1074/jbc.M112.440362 23612974 PMC3682540

[28] Leu CH, Yang ML, Chung NH, Huang YJ, Su YC, Chen YC, Lin CC, Shieh GS, Chang MY, Wang SW, Chang Y, Chao J, Chao L, Wu CL, Shiau AL. 2015. Kallistatin ameliorates influenza virus pathogenesis by inhibition of kallikrein-related peptidase 1-mediated cleavage of viral hemagglutinin. Antimicrob Agents Chemother 59:5619–5630. doi:10.1128/AAC.00065-15 26149981 PMC4538499

[29] Magnen M, Gueugnon F, Guillon A, Baranek T, Thibault VC, Petit-Courty A, de Veer SJ, Harris J, Humbles AA, Si-Tahar M, Courty Y. 2017. Kallikrein-related peptidase 5 contributes to H3N2 influenza virus infection in human lungs. J Virol 91:e00421-17. doi:10.1128/JVI.00421-17 28615200 PMC5533929

[30] Cerqueira C, Samperio Ventayol P, Vogeley C, Schelhaas M. 2015. Kallikrein-8 proteolytically processes human papillomaviruses in the extracellular space to facilitate entry into host cells. J Virol 89:7038–7052. doi:10.1128/JVI.00234-15 25926655 PMC4473586

[31] Jones M, Dry IR, Frampton D, Singh M, Kanda RK, Yee MB, Kellam P, Hollinshead M, Kinchington PR, O’Toole EA, Breuer J. 2014. RNA-seq analysis of host and viral gene expression highlights interaction between varicella zoster virus and keratinocyte differentiation. PLoS Pathog 10:e1003896. doi:10.1371/journal.ppat.1003896 24497829 PMC3907375

[32] Milewska A, Falkowski K, Kulczycka M, Bielecka E, Naskalska A, Mak P, Lesner A, Ochman M, Urlik M, Diamandis E, Prassas I, Potempa J, Kantyka T, Pyrc K. 2020. Kallikrein 13 serves as a priming protease during infection by the human coronavirus HKU1. Sci Signal 13:eaba9902. doi:10.1126/scisignal.aba9902 33234691 PMC7857416

[33] Shaw JLV, Diamandis EP. 2007. Distribution of 15 human kallikreins in tissues and biological fluids. Clin Chem 53:1423–1432. doi:10.1373/clinchem.2007.088104 17573418

[34] Lilja H. 1985. A kallikrein-like serine protease in prostatic fluid cleaves the predominant seminal vesicle protein. J Clin Invest 76:1899–1903. doi:10.1172/JCI112185 3902893 PMC424236

[35] Zang RC, Castro MFG, McCune BT, Zeng QR, Rothlauf PW, Sonnek NM, Liu ZM, Brulois KF, Wang X, Greenberg HB, Diamond MS, Ciorba MA, Whelan SPJ, Ding SY. 2020. TMPRSS2 and TMPRSS4 promote SARS-CoV-2 infection of human small intestinal enterocytes. Sci Immunol 5. doi:10.1126/sciimmunol.abc3582 PMC7285829 32404436

[36] Hou YP, Yu T, Wang TY, Ding Y, Cui Y, Nie HG. 2022. Competitive cleavage of SARS-CoV-2 spike protein and epithelial sodium channel by plasmin as a potential mechanism for COVID-19 infection. Am J Physiol-Lung Cellular Mole Physiol 323:L569–L577. doi:10.1152/ajplung.00152.2022 PMC9639761 36193902

[37] Yousef GM, Chang A, Diamandis EP. 2000. Identification and characterization of KLK-L4, a new kallikrein-like gene that appears to be down-regulated in breast cancer tissues. J Biol Chem 275:11891–11898. doi:10.1074/jbc.275.16.11891 10766816

[38] Darnell JE Jr, Kerr IM, Stark GR. 1994. Jak-STAT pathways and transcriptional activation in response to IFNs and other extracellular signaling proteins. Science 264:1415–1421. doi:10.1126/science.8197455 8197455

[39] Soler ZM, Schlosser RJ, Mulligan JK, Smith TL, Mace JC, Ramakrishan VR, Norris‐Caneda K, Bethard JR, Ball LE. 2021. Olfactory cleft mucus proteome in chronic rhinosinusitis: a case‐control pilot study. Int Forum Allergy Rhinol 11:1162–1176. doi:10.1002/alr.22743 33275311 PMC8670410

[40] Workman AD, Nocera AL, Mueller SK, Otu HH, Libermann TA, Bleier BS. 2019. Translating transcription: proteomics in chronic rhinosinusitis with nasal polyps reveals significant discordance with messenger RNA expression. Int Forum Allergy Rhinol 9:776–786. doi:10.1002/alr.22315 30775848

[41] Yoshikawa K, Wang H, Jaen C, Haneoka M, Saito N, Nakamura J, Adappa ND, Cohen NA, Dalton P. 2018. The human olfactory cleft mucus proteome and its age-related changes. Sci Rep 8:17170. doi:10.1038/s41598-018-35102-2 30464187 PMC6249231

[42] Zheng N, Liu S, Chen J, Xu Y, Cao W, Lin J, Lu G, Zhang G. 2024. SARS-CoV-2 NSP2 as a potential delivery vehicle for proteins. Mol Pharm 21:1149–1159. doi:10.1021/acs.molpharmaceut.3c00680 38288708

[43] Ju X, Zhu Y, Wang Y, Li J, Zhang J, Gong M, Ren W, Li S, Zhong J, Zhang L, Zhang QC, Zhang R, Ding Q. 2021. A novel cell culture system modeling the SARS-CoV-2 life cycle. PLoS Pathog 17:e1009439. doi:10.1371/journal.ppat.1009439 33711082 PMC7990224

[44] Andrade D, Assis DM, Santos JAN, Alves FM, Hirata IY, Araujo MS, Blaber SI, Blaber M, Juliano MA, Juliano L. 2011. Substrate specificity of kallikrein-related peptidase 13 activated by salts or glycosaminoglycans and a search for natural substrate candidates. Biochimie 93:1701–1709. doi:10.1016/j.biochi.2011.05.037 21689719

[45] Buchrieser J, Dufloo J, Hubert M, Monel B, Planas D, Rajah MM, Planchais C, Porrot F, Guivel-Benhassine F, Van der Werf S, Casartelli N, Mouquet H, Bruel T, Schwartz O. 2020. Syncytia formation by SARS-CoV-2-infected cells. EMBO J 39:e106267. doi:10.15252/embj.2020106267 33051876 PMC7646020

[46] Xia S, Liu M, Wang C, Xu W, Lan Q, Feng S, Qi F, Bao L, Du L, Liu S, Qin C, Sun F, Shi Z, Zhu Y, Jiang S, Lu L. 2020. Inhibition of SARS-CoV-2 (previously 2019-nCoV) infection by a highly potent pan-coronavirus fusion inhibitor targeting its spike protein that harbors a high capacity to mediate membrane fusion. Cell Res 30:343–355. doi:10.1038/s41422-020-0305-x 32231345 PMC7104723

[47] Braga L, Ali H, Secco I, Chiavacci E, Neves G, Goldhill D, Penn R, Jimenez-Guardeño JM, Ortega-Prieto AM, Bussani R, Cannatà A, Rizzari G, Collesi C, Schneider E, Arosio D, Shah AM, Barclay WS, Malim MH, Burrone J, Giacca M. 2021. Drugs that inhibit TMEM16 proteins block SARS-CoV-2 spike-induced syncytia. Nature 594:88–93. doi:10.1038/s41586-021-03491-6 33827113 PMC7611055

[48] Zhang Z, Zheng Y, Niu Z, Zhang B, Wang C, Yao X, Peng H, Franca DN, Wang Y, Zhu Y, et al.. 2021. SARS-CoV-2 spike protein dictates syncytium-mediated lymphocyte elimination. Cell Death Differ 28:2765–2777. doi:10.1038/s41418-021-00782-3 33879858 PMC8056997

[49] Papa G, Mallery DL, Albecka A, Welch LG, Cattin-Ortolá J, Luptak J, Paul D, McMahon HT, Goodfellow IG, Carter A, Munro S, James LC. 2021. Furin cleavage of SARS-CoV-2 spike promotes but is not essential for infection and cell-cell fusion. PLoS Pathog 17:e1009246. doi:10.1371/journal.ppat.1009246 33493182 PMC7861537

[50] Qi LS, Larson MH, Gilbert LA, Doudna JA, Weissman JS, Arkin AP, Lim WA. 2013. Repurposing CRISPR as an RNA-Guided Platform for Sequence-Specific Control of Gene Expression. Cell 152:1173–1183. doi:10.1016/j.cell.2013.02.022 23452860 PMC3664290

[51] Gruba N, Bielecka E, Wysocka M, Wojtysiak A, Brzezińska-Bodal M, Sychowska K, Kalińska M, Magoch M, Pęcak A, Falkowski K, Wiśniewska M, Sąsiadek L, Płaza K, Kroll E, Pejkovska A, Rehders M, Brix K, Dubin G, Kantyka T, Potempa J, Lesner A. 2019. Development of chemical tools to monitor human kallikrein 13 (KLK13) activity. Int J Mol Sci 20:1557. doi:10.3390/ijms20071557 30925705 PMC6479877

[52] Delaveris CS, Webster ER, Banik SM, Boxer SG, Bertozzi CR. 2020. Membrane-tethered mucin-like polypeptides sterically inhibit binding and slow fusion kinetics of influenza A virus. Proc Natl Acad Sci U S A 117:12643–12650. doi:10.1073/pnas.1921962117 32457151 PMC7293601

[53] Iverson E, Griswold K, Song D, Gagliardi TB, Hamidzadeh K, Kesimer M, Sinha S, Perry M, Duncan GA, Scull MA. 2022. Membrane-tethered mucin 1 is stimulated by interferon and virus infection in multiple cell types and inhibits influenza a virus infection in human airway epithelium. MBio 13:e0105522. doi:10.1128/mbio.01055-22 35699372 PMC9426523

[54] Wallace LE, Liu M, van Kuppeveld FJM, de Vries E, de Haan CAM. 2021. Respiratory mucus as a virus-host range determinant. Trends Microbiol 29:983–992. doi:10.1016/j.tim.2021.03.014 33875348 PMC8503944

[55] Kim H, Kang Y, Kim S, Park D, Heo S-Y, Yoo J-S, Choi I, N MPA, Ahn J-W, Yang J-S, Bak N, Kim KK, Lee J-Y, Choi YK. 2024. The host protease KLK5 primes and activates spike proteins to promote human betacoronavirus replication and lung inflammation. Sci Signal 17:eadn3785. doi:10.1126/scisignal.adn3785 39163389

[56] Liang YS, Zhang GG, Li QH, Han L, Hu XY, Guo Y, Tao WY, Zhao XM, Guo MZ, Gan TY, Tong YM, Xu YF, Zhou Z, Ding Q, Wei WS, Zhong J. 2021. TRIM26 is a critical host factor for HCV replication and contributes to host tropism. Sci Adv 7. doi:10.1126/sciadv.abd9732 PMC7793585 33523994

[57] Pan T, Chen R, He X, Yuan Y, Deng X, Li R, Yan H, Yan S, Liu J, Zhang Y, Zhang X, Yu F, Zhou M, Ke C, Ma X, Zhang H. 2021. Infection of wild-type mice by SARS-CoV-2 B.1.351 variant indicates a possible novel cross-species transmission route. Sig Transduct Target Ther 6:420. doi:10.1038/s41392-021-00848-1 PMC8669038 34907154

