# Supplementary figures and images for: Nasal mucus-derived KLK13 restricts SARS-CoV-2 infection via proteolytic cleavage of spike
Source: mBio. 2025 Oct 20;16(11):e02051-25. doi: 10.1128/mbio.02051-25 (PMC12607584; doi:10.1128/mbio.02051-25)

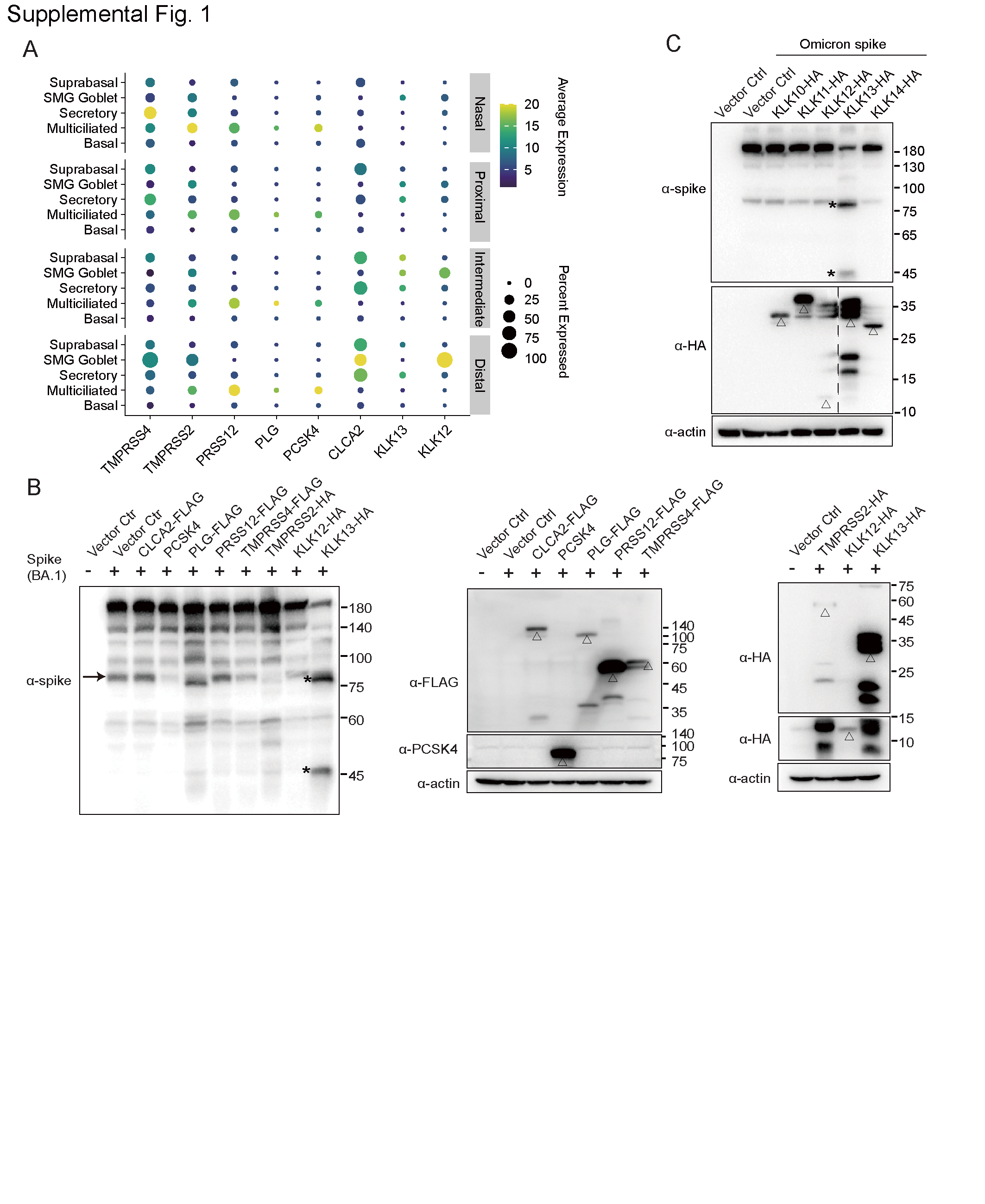

Supplement: Fig. S1 — KLK13 cleaves the spike of SARS-CoV-2 (BA.1 strain). [file mbio.02051-25-s0001.tif]

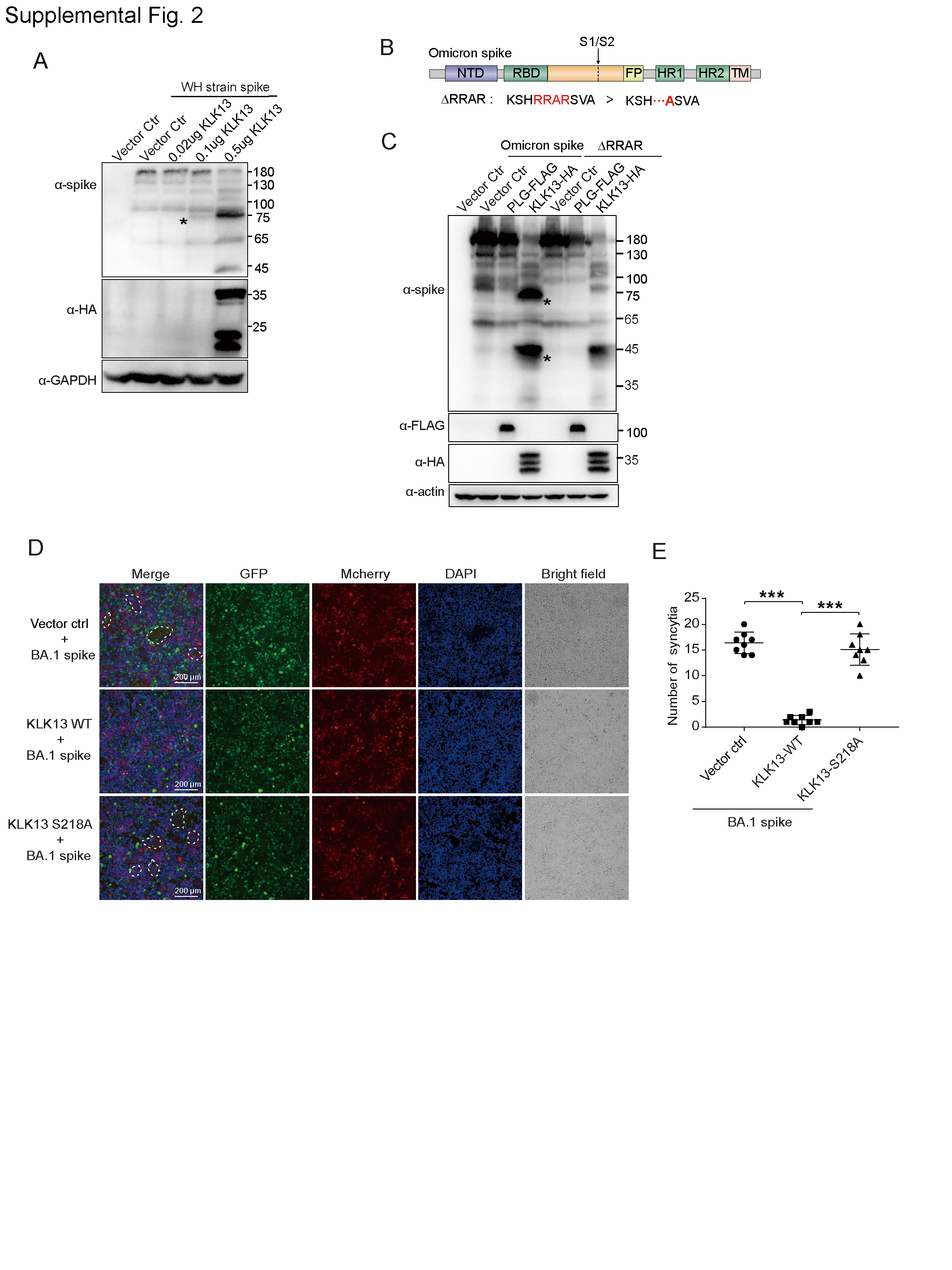

Supplement: Fig. S2 — Deletion of RRAR affected KLK13-mediated cleavage of SARS-CoV-2 spike. [file mbio.02051-25-s0002.tif]

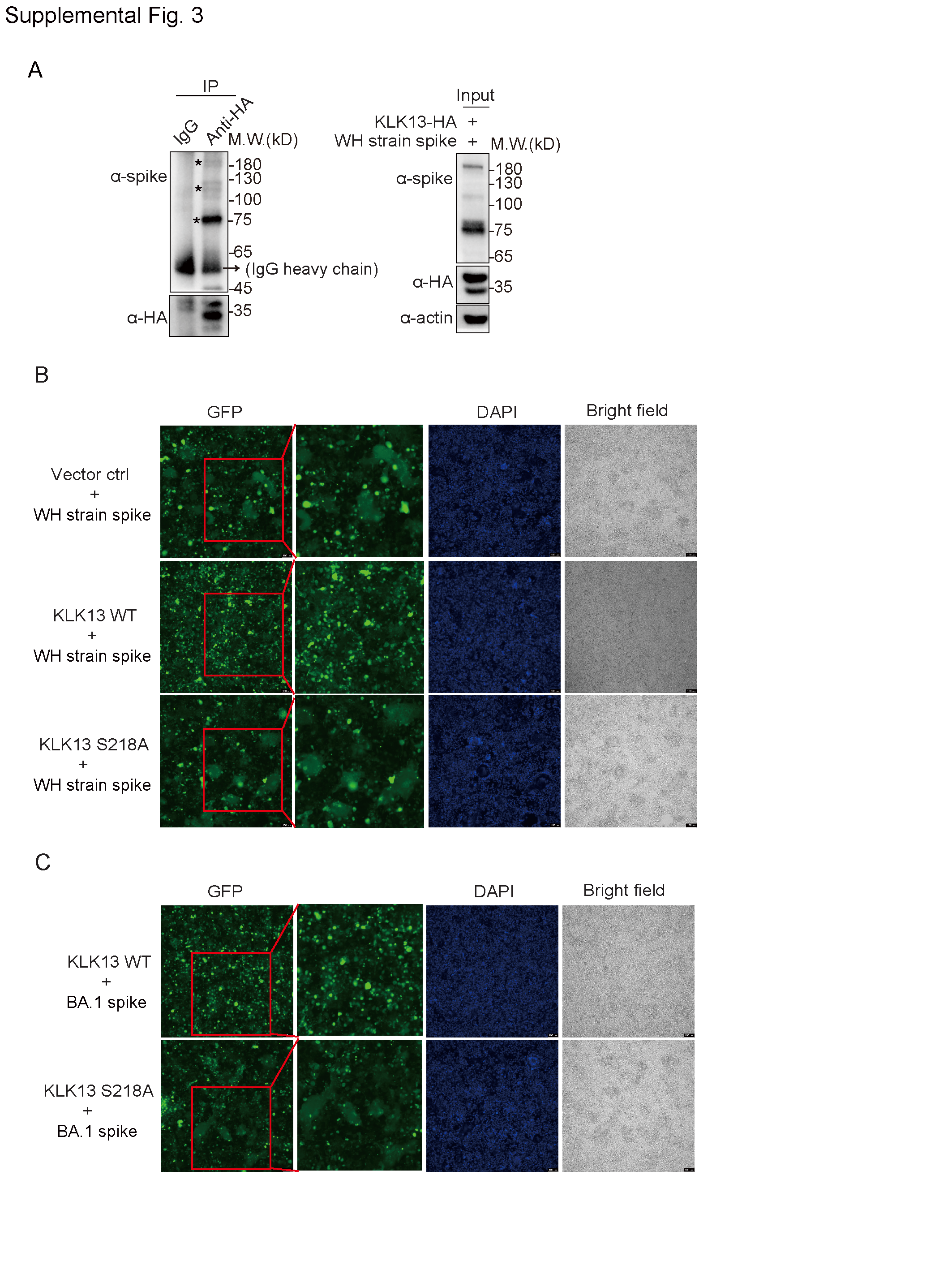

Supplement: Fig. S3 — KLK13 interacted with the SARS-CoV-2 spike protein and inhibited spike protein-mediated cell-cell fusion. [file mbio.02051-25-s0003.tif]

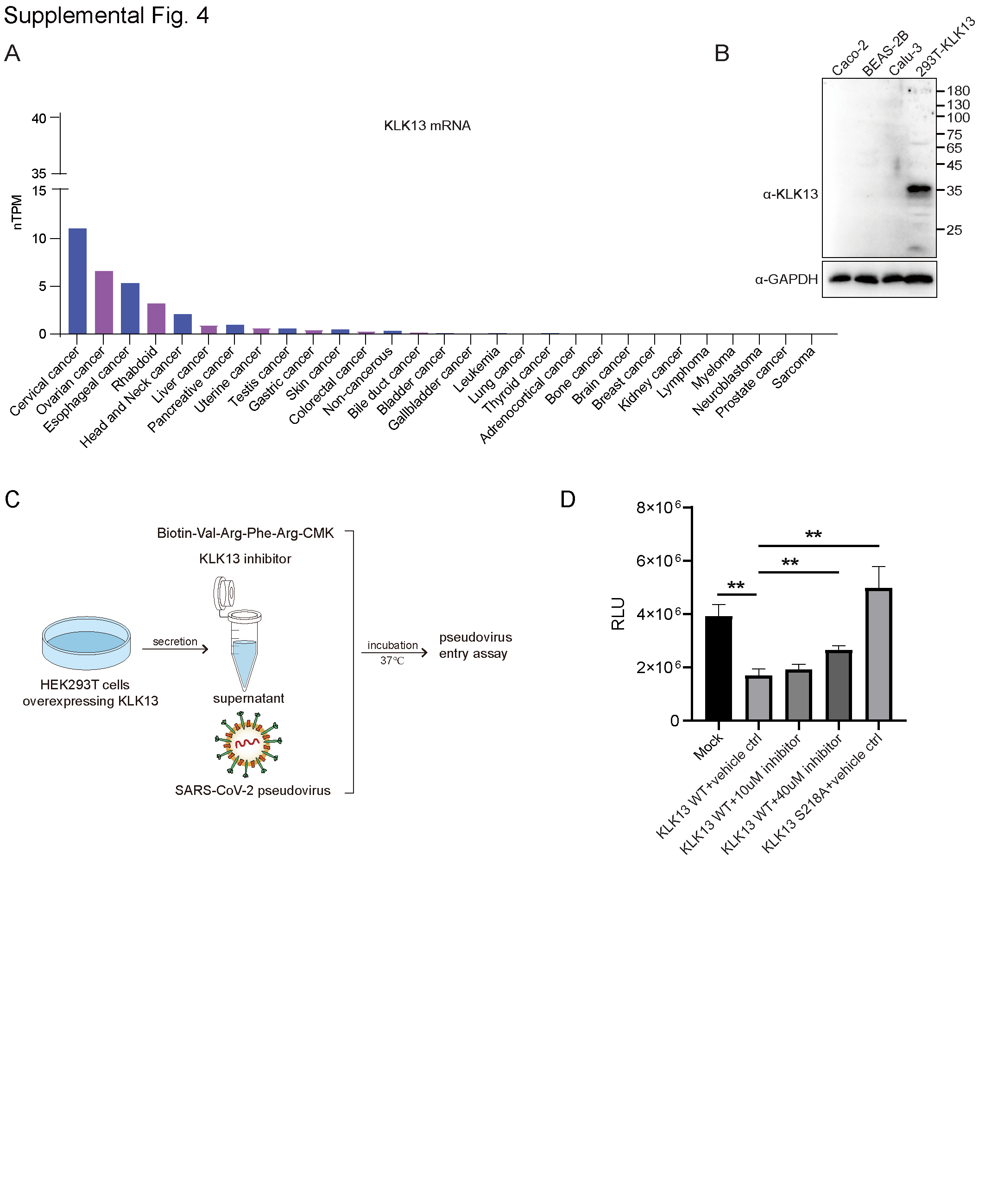

Supplement: Fig. S4 — Low expression of KLK13 in different cell lines. [file mbio.02051-25-s0004.tif]

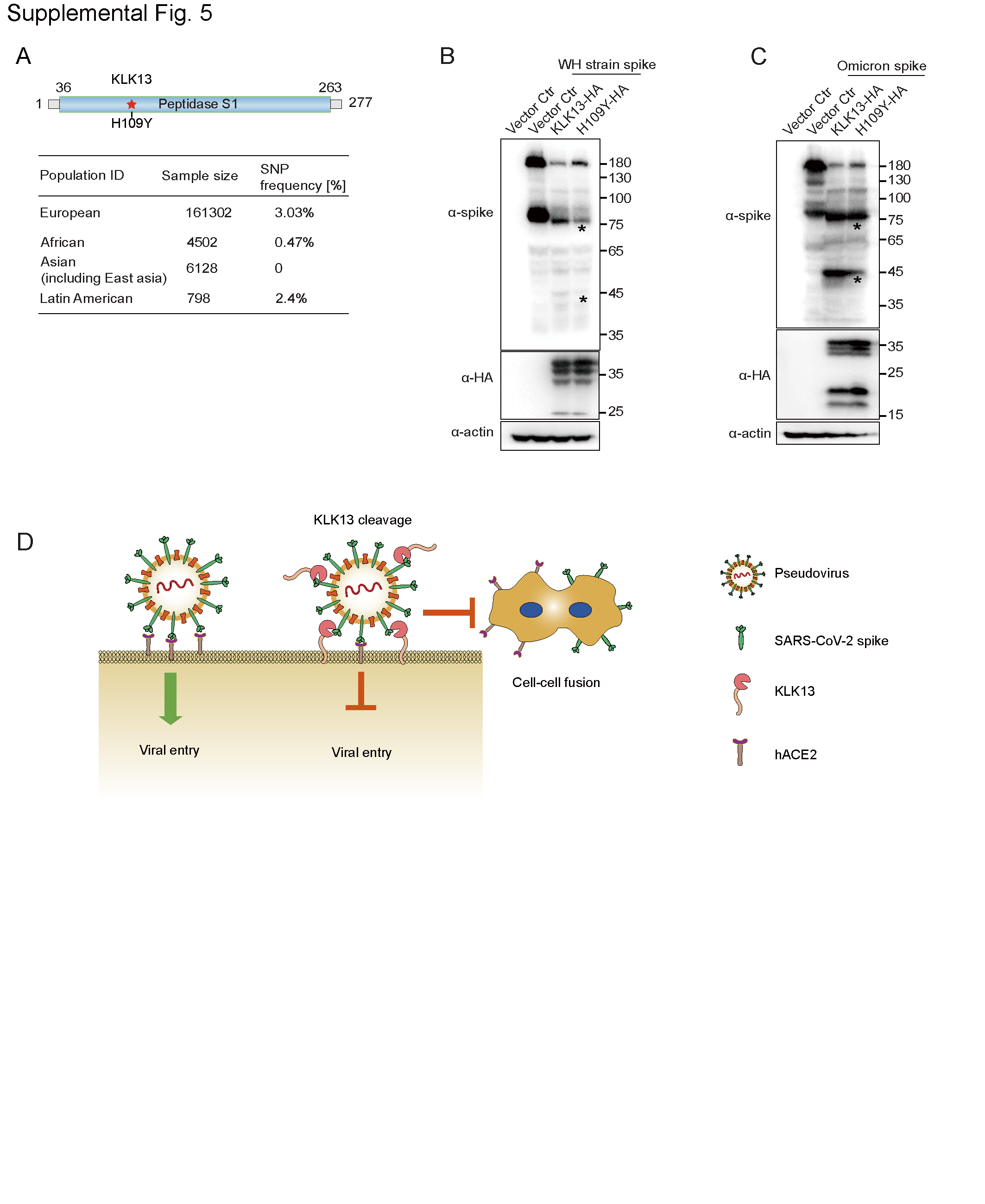

Supplement: Fig. S5 — KLK13 polymorphism H109Y slightly reduced the cleavage efficiency on SARS-CoV-2 spike. [file mbio.02051-25-s0005.tif]
