# Supplementary material for: Nasal mucus-derived KLK13 restricts SARS-CoV-2 infection via proteolytic cleavage of spike
Source: mBio. 2025 Oct 20;16(11):e02051-25. doi: 10.1128/mbio.02051-25 (PMC12607584; doi:10.1128/mbio.02051-25)
Supplement: Legends — Supplemental figure legends. [file mbio.02051-25-s0006.docx]

**Supplemental Materials**

**Supplemental Fig. 1. KLK13 cleaves the spike of SARS-CoV-2 (BA.1 strain).** **(A)** The expression levels of eight genes in annotated cell types of the respiratory tract epithelium were evaluated using single-cell RNA sequencing analysis. Dot size indicates the percent of expressing cells, while colour indicates the average expression level. **(B)** Expression plasmid for Omicron (BA.1 strain) spike together with CLCA2-FLAG, PCSK4, PLG-FLAG, PRSS12-FLAG, TMPRSS4-FLAG, TMPRSS2-HA, KLK12-HA, KLK13-HA, or empty vector were cotransfected into HEK293T cells for 48 h. The cells were lysed and analysed using immunoblotting assay. This experiment was performed in two biological replicates. **(C)** The plasmids expressing Omicron (BA.1 strain) spike together with HA-tagged KLK10, KLK11, KLK12, KLK13, KLK14 or empty vector were cotransfected into HEK293T cells. Forty-eight hours later, the cells were lysed and subjected to immunoblotting analysis. This experiment was performed in three biological replicates. The asterisks (**B-C**) indicate the two cleaved fragments of spike protein. The arrowheads indicate the S2 subunit. The triangles indicate the proteases overexpressed in the cells.

**Supplemental Fig. 2. Deletion of RRAR affected KLK13 mediated cleavage of SARS-CoV-2 spike.** **(A)** HEK293T cells were cotransfected with expression plasmids for SARS-CoV-2 WH strain spike and different quantities of KLK13-WT. Cell lysates were prepared and subjected to Western blotting analysis. This experiment was performed in three biological replicates. **(B)** Schematic representation of the domains of SARS-CoV-2 Omicron (BA.1) spike and its mutant with RRAR deletion (∆RRAR). **(C)** Omicron (BA.1) spike or ∆RRAR plasmid was cotransfected together with KLK13-HA or PLG-FLAG overexpression vector, respectively, into HEK293T cells. The cells were lysed after 48 h and analysed by immunoblotting assay. The asterisks indicate the cleaved fragments of spike protein. **(D)** HEK293T cells were cotransfected with Omicron (BA.1) spike plasmid along with KLK13-HA, KLK13^S218A^ mutant or empty vector, respectively. Five hours later, the cells were detached and cocultured with HEK293T cells expressing ACE2 and GFP at a ratio of 1:1. The cells were fixed 36 h post coculture and subsequently stained with DAPI. Fluorescence and bright field images were obtained using immunofluorescence microscopy. The syncytium was depicted by merged red-green colour and circled by dashed lines. **(E)** Quantification of the syncytia shown in (D). Syncytium number was pooled from eight microscope fields for each experiment. Student’s *t* test was used to perform statistical analysis (n=8). This experiment was performed in two biological replicates.

**Supplemental Fig. 3. KLK13 interacted with the SARS-CoV-2 spike protein and inhibited spike protein-mediated cell-cell fusion. (A)** HEK293T cells were cotransfected with expression vectors for SARS-CoV-2 WH strain spike and KLK13-HA. The cellular lysates were subjected to immunoprecipitation using protein A/G agarose beads conjugated with specific IgG against HA or normal IgG. The precipitates and lysates were blotted with specific antibodies. HEK293T-ACE2-GFP cells were cotransfected with plasmids encoding SARS-CoV-2 (WH strain) spike protein **(B)** or Omicron (BA.1 strain) spike protein **(C)** together with KLK13-WT, KLK13^S218A^ or empty vector for 48 hours. Immunofluorescence microscopy was used to capture fluorescence (GFP) and bright field images to assess syncytia formation.

**Supplemental Fig. 4. Low Expression of KLK13 in different cell lines.** **(A)** The expression levels of *KLK13* mRNA in various cell lines, according to the data of The Human Protein Atlas. **(B)** Evaluation of expression levels of KLK13 in Caco-2, BEAS-2B and Calu-3 cells by immunoblotting analysis with specific antibodies, while HEK293T-KLK13 cells served as a positive control. **(C)** Schematic diagram of pseudovirus entry assays with KLK13 inhibitor. **(D)** Supernatants containing secreted KLK13 were pre-incubated with different concentrations of KLK13 inhibitor (0 μM, 10 μM and 40 μM) and pseudoviruses at 37 ℃ overnight. The KLK13 and inhibitor treated pseudoviruses were prepared for the pseudovirus entry assay. The entry of pseudovirus was quantified through measuring luciferase activity (n=3). This experiment was performed in two biological replicates.

**Supplemental Fig. 5. KLK13 polymorphism H109Y slightly reduced the cleavage efficiency on SARS-CoV-2 spike. (A)** Graphical representation of KLK13 protein and the mutant site located in peptidase S1 domain (upper panel). The frequency of SNP (rs34089525) in *KLK13* of indicated human populations is shown in table (lower panel). SARS-CoV-2 (WH strain) spike **(B)** or (BA.1 strain) spike **(C)** expression plasmid was transfected into HEK293T cells along with HA tagged wild-type KLK13, KLK13^H109Y^, or empty vector. The cells were prepared for immunoblotting analysis after 48 h of transfection. The asterisks indicate the cleaved fragments of spike protein. These experiments were performed in two biological replicates. **(D)** Schematic illustration of the impact of KLK13 on SARS-CoV-2 entry and cell-cell fusion. Spike cleavage by membrane-bound or secretory KLK13 inhibits virus entry and spike induced cell-cell fusion.
